# Current status of drug screening and disease modelling in human pluripotent stem cells

**DOI:** 10.1002/bies.201200053

**Published:** 2012-08-08

**Authors:** Divya Rajamohan, Elena Matsa, Spandan Kalra, James Crutchley, Asha Patel, Vinoj George, Chris Denning

**Affiliations:** Department of Stem Cells, Tissue Engineering & Modelling, Centre for Biomolecular Sciences, University of NottinghamNottingham, UK

**Keywords:** automation, cardiomyocytes, drug safety assessment, human embryonic stem cells, human induced pluripotent stem cells

## Abstract

The emphasis in human pluripotent stem cell (hPSC) technologies has shifted from cell therapy to in vitro disease modelling and drug screening. This review examines why this shift has occurred, and how current technological limitations might be overcome to fully realise the potential of hPSCs. Details are provided for all disease-specific human induced pluripotent stem cell lines spanning a dozen dysfunctional organ systems. Phenotype and pharmacology have been examined in only 17 of 63 lines, primarily those that model neurological and cardiac conditions. Drug screening is most advanced in hPSC-cardiomyocytes. Responses for almost 60 agents include examples of how careful tests in hPSC-cardiomyocytes have improved on existing in vitro assays, and how these cells have been integrated into high throughput imaging and electrophysiology industrial platforms. Such successes will provide an incentive to overcome bottlenecks in hPSC technology such as improving cell maturity and industrial scalability whilst reducing cost.

## Introduction

When human embryonic stem cells (hESCs) were first isolated from blastocyst stage embryos in 1998 [1], many researchers believed that within 10–15 years the technology would be sufficiently advanced to allow cell replacement of tissues damaged by injury, disease or aging. Within the next few years, approximately 1200 hESC lines had been derived (http://www.umassmed.edu/iscr/index.aspx) and it became possible to produce human induced pluripotent stem cells (hiPSCs) by reprogramming somatic cells with just four genetic factors [2, 3]. This provided a considerable resource of human pluripotent stem cells (hPSCs) that could be propagated during long-term culture and yet be differentiated to a variety of lineages representative of the three embryonic germ layers [4]. Clinically relevant cell types included cardiomyocytes and blood lineages (mesoderm), hepatocytes and pancreatic lineages (endoderm) and neural and dermal lineages (ectoderm).

An unexpected hurdle was that methods to culture and differentiate hPSCs were inefficient and labour-intensive [5]. Improvements in cell passaging and commercial provision of defined culture media (e.g. mTeSR [6], Stem Cell Technologies; StemPro, Invitrogen [7]) reduced the labour required by individual labs. Nevertheless, even defined media are susceptible to considerable batch to batch variability, probably due to growth factor manufacture inconstancies or degradation of the growth factors during storage. Growth substrate is another source of variability. hPSCs are typically grown on biological substrates such as human or mouse feeder cells, extracted matrices (e.g. Matrigel) or recombinant proteins (e.g. laminin, collagen, fibronectin and vitronectin), all of which are expensive, variable and/or labile [8]. Although synthetic substrates that support pluripotency in defined culture media are an exciting development [9, 10], further refinement is needed. For example, hPSCs can be maintained on Corning® Synthemax™ substrates in mTeSR culture medium [10] but a single 6-well plate costs $75 and passaging cells requires manual scraping, which is not amenable to scaled automation. For differentiation, it is now very encouraging that protocols exist to yield purities in excess of 50–70% for many cell types. However, the diversity of methods published for each differentiated cell lineage [11] belies the challenge of successfully reproducing protocols between different hPSC lines and labs.

## The use of hPSC-derivatives in cell replacement therapy faces challenges

In addition to the difficulties discussed above, cell transplantation also brings many other hurdles to the fore. These include regulatory and ethical issues, whether cells survive, engraft in the correct location and function after delivery, whether patients can be recruited successfully, and the costs associated with clinical trials. The first to transplant hESC derivatives into humans in 2009 [12], Geron Corporation had to convince the Food and Drug Administration (FDA) that their GRNOPC1 neural progenitor cell line was suitable for transplantation into patients with thoracic spinal cord injury with a 22,000 page document detailing the in vitro and preclinical characterisation that had been performed over many years. Although no adverse events were recorded after GRNOPC1 transplantation and the Regulators approved progression to a Phase II trial, spiralling costs led Geron to abandon their entire hESC programme in late 2011. Many researchers viewed this as a major setback for clinical translation of hPSC-based cell replacement therapies. However, Advanced Cell Technology (ACT) recently received FDA approval for clinical trials to treat macular degeneration with hESC-derived retinal pigment epithelium (RPE) cells [13] and these trials will be watched with interest. Nevertheless, it is sobering that after 14 years of research, there is only one active clinical trial using hPSC-derivatives (see clinicaltrials.gov). It is now becoming accepted that a faster route to realising the potential of hPSCs and their differentiated derivatives is through in vitro application, particularly in drug safety assessment and in providing novel models of genetic disease.

## Human conditions are not always reflected in animal models because of species differences

Although in vitro disease modelling could theoretically be realised by harvesting primary cells from healthy donors or those carrying a relevant genetic condition, for many cell types this is not a realistic option. For example, harvesting heart tissue on an industrial scale is limited by suitable donors, lack of proliferation of cardiomyocytes, variability in preparation, disease state and cell viability. These problems are particularly pronounced if the cells are sourced from cadavers. Consequently, there is considerable reliance on material derived from animals. Mice are most commonly used for modelling disease because of the relative ease of precisely manipulating the genome by gene targeted homologous recombination [14]. However, there are major differences in the gene expression and physiology between animals and humans, from the single cell level up to the whole animal. The beat rate of the mouse heart is approximately ten times faster than the human (500 bpm vs. 60 bpm) but it has an electrocardiogram duration 5–10 times shorter (450 milliseconds vs. 50–100 milliseconds) [15]. Increases in heart rate are associated with increased force of contraction in humans but decreased force in mice [16]. Whereas repolarisation of the mouse cardiomyocytes is driven primarily by *I*_to_, *I*_K,slow1_, *I*_K,slow2_, *I*_SS_ ion channels, this function is provided by the potassium channels, *I*_Kr_ and *I*_Kr_ in human cells [15]. There are species differences in the role of the regulatory molecule, phospholamban [15], and expression of structural genes also varies. In humans, expression of alpha and beta myosin heavy chains (α-/β-MHC) locates to the atria and ventricles, respectively [17], but in the mouse αMHC is expressed in both locations [18]. The surface marker, SIRPA, is expressed on cardiomyocytes from human but not mouse hPSCs, and so only the human cells can be enriched by fluorescence or magnetic activated cell sorting [19].

Such differences mean that extrapolation from mouse to human can be misleading. In humans, long QT syndrome (LQTS) type 1 and type 2 are caused by mutations that affect function of *I*_Ks_ and *I*_Kr_, respectively, and can lead to palpitations, syncope (fainting), seizures and sudden cardiac death [20]. Since repolarisation of the mouse heart does not rely on these channels, this animal cannot be used to model the conditions. Outside the cardiovascular system, the survival motor neuron 2 gene (SMN2) gene is implicated in development of spinal muscular atrophy in humans, but this gene is not present in mice, flies and worms [21]. The gene sequence of α-synuclein found in healthy wildtype mice and rats can confer Parkinson's disease in humans [22]. The ontology of organs affected by cystic fibrosis in humans differs markedly from that in mice [23]. Such observations have prompted development of novel in vitro human-based systems for studying human genetic disease.

## Development of hPSC-based models of human genetic disease is needed

Human pluripotent stem cells have the potential to play a major role in providing models of genetic disease. Early efforts were directed towards using hESCs, and there are about a dozen examples of where cases in which this has been achieved [24]. Lines carrying myotonic dystrophy type 1, cystic fibrosis and Huntington disease have been derived by isolating hESCs from pre-implantation genetic diagnosis (PGD) embryos [25]. However, PGD screens for only a limited number of genetic conditions, few scientists have access to these facilities and the use of embryos (even those that harbour detrimental genetic lesions) is ethically sensitive in many countries. Alternatively, gene targeting has been used to inactivate genes, such as HPRT1 in male hESCs, to produce an in vitro model of the metabolic disorder Lesch Nyhan syndrome [26]. However, while manipulation of the hPSC genome has become more routine in the last few years [27], engineering specific polymorphisms, deletions or amplifications is time consuming, requires a reasonable level of skill, and becomes increasingly challenging proportionate with the number and complexity of modifications required, even when nuclease-based methods are used [28].

**Figure 1:**
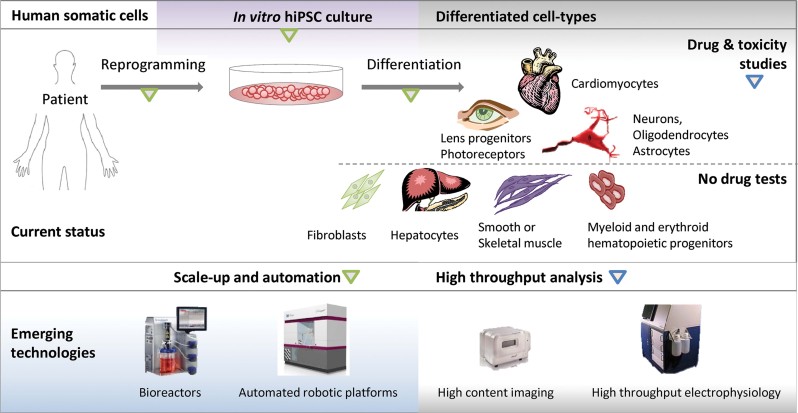
Current status and emerging technologies in disease modelling and drug screening for hiPSC-based models of human genetic disease. hiPCS-based models of human disease affecting the heart, smooth muscle, skeletal muscle, skin, central nervous system (CNS), liver, blood and eye have been generated. However, only those affecting the heart, CNS and eye have been used to evaluate the effects of drug treatment. Emerging technologies for scale-up, automation and high throughput analysis will enable use of hiPSC-disease models for drug discovery and safety evaluation in an industrial setting. Green and blue arrows show processes amenable to scale-up and automation, or high-content imaging and electrophysiology analysis.

In contrast, hiPSC technology is readily accessible, and has the potential to revolutionise in vitro disease modelling (Table 1; Fig. 1). It is relatively straightforward for scientists to establish collaborations with clinicians who care for patients with a particular genetic condition, and the ethical frameworks for informed patient consent are commonplace within most universities and industrial settings. Many commercial providers of stem cell reagents now offer complete off-the-shelf kits to progress from patient sample to reasonably well characterised hiPSC lines. Consequently, less than 5 years after the first report of reprogramming somatic cells [3], 63 hiPSC models have been produced for 43 diseases affecting the heart, smooth muscle, skeletal muscle, immune system, skin, central nervous system, blood and eye, as well as imprinting, metabolic and multi-organ disorders (Table 1). It can be expected that the number of hiPSC lines available will rise exponentially over the next few years.

**Table 1:** Disease-specific human induced pluripotent stem cells: characterisation and use in drug screening

Category	Disorder	Gene	Method	Phenotype characterisation assays	Drug treatment	Effect	Ref.
Cardiac	Long QT-syndrome type 1 (LQT1)	*KCNQ1*	OSKC retrovirus	Prolonged APD in atrial and ventricular cardiomyocytes	Isoprenaline (100 nM), propranolol (200 nM)	↑ BR, caused EADs	[81]
						Corrected EADs	
	Long QT-syndrome type 2 (LQT2)	*KCNH2*	OSNL lentivirus	Prolonged FPD and APD in atrial and ventricular cardiomyocytes, reduction in *I*_kr_ current	Isoprenaline (100 nM)	↓ BR, caused EADs	[34]
					Nadolol (10 µM), propranolol (200 nM)	Corrected EADs	
					E4031 (1 µM)	↑ FPD/APD, caused EADs	
					Nicorandil (20 µM)	↓ FPD/APD, corrected EADs	
					PD-118057 (3 µM)	↓ FPD/APD	
			OSK retrovirus		E4031 (500 nM), Cisapride (N/S)	↑ FPD/APD, caused arrhythmogenesis	[38]
					Nifedipine (1 mM),	↓ FPD/APD, corrected EADs	
					Pinacidil (1 mM)		
					Ranolazine (15–50 mM)	Reduced arrhythmogenesis	
			OSKC retrovirus	Asymptomatic carrier with LQT2 family history used to diagnose LQT2 as hiPSC-cardiomyocytes showed prolonged FPD/APD	Sotalol (0.8–19.4 µM), E4031 (1 µM)	↑ FPD/APD	[82]
					Erythromycin (1.5–16 µM), cisapride (40–330 nM)	None	
	Catecholaminergic polymorphic ventricular tachycardia type 1 (CPVT1)	*RYR2*	]OSKC retrovirus	Elevated diastolic Ca(2+) concentrations, reduced SR Ca(2+) content, increased susceptibility to DADs and arrhythmias after catecholaminergic stimulation	Isoprenaline (1 µM)	↑ BR, caused DADs	[83]
					Forskolin (5 µM), 8-Br-cAMP (100 µM)	↑ Cytosolic cAMP and abolished Ca(2+)-release events after repolarisation	
			N/A		Dantrolene (N/A)	Restored normal Ca(2+) spark properties and prevented arrhythmogenesis	[35]
	Timothy syndrome (TS)	*CACNA1C*	OSKC retrovirus	Irregular cardiac myocyte contraction, excess Ca(2+) influx, prolonged APD, irregular electrical activity, abnormal calcium transients	Roscovitine (33.3 µM)	↑ Ca(V)1.2 voltage-dependent inactivation, restored electrical and Ca(2+) signalling properties	[36]
			OSKC retrovirus	Abnormal expression of tyrosine hydroxylase and increased production of norepinephrine and dopamine in neurons	Roscovitine (N/S)	Reversed abnormal phenotype	[37]
	LEOPARD syndrome (includes Noonan syndrome)	*PTPN11, RAF1, SHOC2*	OSKC retrovirus	Increased sarcomeric organisation and preferential localisation of NFATC4 in the nucleus, which correlate with potential hypertrophic state. Study of molecular insights into disease mechanism	None	None	[33]
Smooth muscle	Hutchinson-Gilford progeria syndrome (HGPS)	*LMNA*	OSKC retrovirus	Premature senescence in smooth muscle cells. DNAPKcs identified as progerin target, therefore uncovering disease pathogenesis	Lentiviral anti-progerinshRNA	Phenotype correction	[84]
			OSKC retrovirus	DNA damage, nuclear abnormalities and calponin-staining inclusion bodies in MSCs, smooth muscle cells and fibroblasts	None	None	[85]
Skeletal muscle	Duchene muscular dystrophy (DMD)	*Dystrophin*	OSKC retrovirus	Genotyping	None	None	[86]
			OSNL lentivirus	Genotyping	None	None	[87]
			OSK retrovirus	Gene-corrected hiPSCs generated using a human artificial chromosomes with complete genomic dystrophin sequence	None	None	[88]
	Becker muscular dystrophy (BMD)	*Dystrophin*	OSKC retrovirus	Genotyping	None	None	[86]
Immune	Adenosine deaminase deficiency-associated severe combined immunodeficiency (ADA-SCID)	*ADA*	OSKC retrovirus	Genotyping	None	None	[86]
	Multiple-sclerosis (MS)	*MHC*	OSKC retrovirus	Differentiation to oligodendrocytes, astrocytes and functional neurons	None	None	[89]
Imprinting	Angelman syndrome	*UBE3A*	OSKCL retrovirus	*UBE3A* paternalimprinting re-established during hiPSC neuronal differentiation	None	None	[90]
	Pradder-Willi		OSKC retrovirus	*UBE3A* maternal imprinting maintained in hiPSCs, reduced expression of disease-associated RNA HBII-85/SNORD11	None	None	[91]
Skin	Recessive dystrophic epidermolysisbullosa (RDEB)	*COL7A1*	OSKC retrovirus	Gene-corrected RDEB hiPSCs expressed Col7 and differentiated to skin	None	None	[92]
Neurological	Spinal muscular atrophy (SMA)	*SMN1*	OSKC retrovirus	Reduced differentiation to motoneurons, abnormal neurite outgrowth. Genetic correction of phenotype by ectopic SMN over-expression	None	None	[93]
			OSNL lentiviral	Deficits in motor neurons, lack of nuclear gems	Valproic acid (1 mM), tobramycin (320 mM)	↑ Number of nuclear gems and SMN protein expression	[21]
	Familial dysautonomia (FD)	*IKBKAP*	OSKC lentivirus	Neurogenic differentiation and migration defects, decreased expression of peripheral neurogenesis and neuronal differentiation markers	Kinetin (N/S)	↓ Mutant *IKBKAP* splice variant, ↑ wild-type transcript, ↑ neuronal differentiation and neuronal marker expression	[94]
					Epigallocatechin, gallate (N/S), tocotrienol (N/S)	None	
	Rett syndrome (RTT)	*MECP2*	OSKC retrovirus	Genotyping and differentiation to neurons	None	None	[95]
			OSKC retrovirus	Reduced synapses and dendritic spine density, smaller soma size, altered calcium signalling and electrophysiological defects in neurons, altered neuronal network signalling	IGF1 (0.01 nM)	↑ Glutamatergic synapses	[32]
					Gentamicin (100 nM)	Enabled expression of full length MeCP2 protein	
					Gabazine (N/S)	↑ Ca(2+) transients	
		*CDKL5*	N/S	Genotyping and differentiation to neurons	None	None	[96]
	Schizophrenia (SCZD)	*DISC1*	OSNLKC + SV40L Episomal	Genotyping and differentiation to neurons	None	None	[97]
		*N/S*	OSKCL tet-inducible lentivirus	Reduced neuronal connectivity, soma outgrowths and PSD95 dendritic protein, altered gene expression profiles implicating Notch signalling, cell adhesion and Slit-Robo-mediated axon guidance in disease pathogenesis	Loxapine (N/S)	Improved neuronal connectivity and gene expression profiles	[29]
					Clozapine, olanzapine, risperidone, thioridazine (N/S)	None	
	Alzheimer's disease (AD)	*PS1, PS2*	OSNLK retrovirus	Increased amyloid Aβ42 secretion in neurons	Compound E (γ-secretase inhibitor XXI; 10–100 nM)	↓ Aβ42 and Aβb40 production	[30]
			OSK retrovirus		Compound W (selective Aβ42-lowering agent; 10–100 µM)	↓ Aβ42:Aβ40 ratio	[98]
	Early onset Alzheimer's disease (AD) in Down syndrome patients	*APP* over-expression due to *Trisomy 21*	N/S	Differentiation to cortical neurons secreting pathogenic hyperphosphorylated tau protein and Aβ42, which formed insoluble amyloid aggregates	γ-Secretase inhibitor (N/S)	↓ Aβ42 and Aβb40 production	[31]
	Parkinson's disease (PD)	*PINK1*	OSKC retrovirus	Genotyping	None	None	[86]
			OSK Cre-excisable lentivirus	Genotyping and differentiation to dopaminergic neurons	None	None	[99]
			OSKC retrovirus	Dopaminergic neurons with impaired Parkin recruitment to mitochondria, increased mitochondrial copy number, upregulation of PGC-1α. Phenotype correction with PINK1 over-expression	None	None	[100]
		*LRRK2 Idiopathic*	OSK retrovirus	Dopaminergic neurons with morphological alterations, reduced neurite numbers, neurite arborisation and increased autophagicvacuolation	None	None	[101]
	Fragile-X syndrome (FXS)	*FMR1*	OSKC retrovirus	hiPSC aberrant neuronal differentiation directly related to epigenetic modification of *FMR1* and loss of FMR protein expression	None	None	[102]
	Friedreich ataxia (FRDA)	*FXN*	OSKC retrovirus	Differentiation to peripheral neurons and cardiomyocytes	None	None	[103]
	Huntington's disease (HD)	*Huntingtin*	OSKC retrovirus	Genotyping	None	None	[86]
			OSKC retrovirus	Differentiation to neurons with elevated caspase activity	None	None	[104]
	Olivopontocerebellar atrophy (OPCA)	*SCA7*	OSKC	Differentiation to neural cells	None	None	[105]
	Autism spectrum disorders (ASDs)	*Multifactorial*	N/A	Differentiation to GABAergic neurons	None	None	[106]
	Amyotrophic lateral sclerosis (ALS)	*SOD1*	OSKC retrovirus	Genotyping, differentiation to motor neurons and glia	None	None	[107]
Metabolic	Gaucher disease type III (GBA)	*GBA*	OSKC retrovirus	Genotyping	None	None	[86]
	Lesch-Nyhan syndrome	*HPRT1*	OSKC retrovirus	Genotyping	None	None	[86]
	Juvenileonset type 1 diabetesmellitus (T1D)	*Multifactorial*	OSKC retrovirus	Genotyping	None	None	[86]
			OSK retrovirus	Differentiation to insulin-producing cells	None	None	[108]
	Type 2 diabetes (T2D)	*Multifactorial*	OSKC retrovirus	Differentiation to insulin-producing islet-like progeny	None	None	[109]
	Alpha1-antitrypsin deficiency (A1ATD)	*A1AT*	OSKC retrovirus	Differentiation to hepatocytes with endoplasmic reticulum aggregates of misfolded α1-antitrypsin	None	None	[110]
	Familial hypercholesterolemia (FH)	*LDLR*		Differentiation to hepatocytes with deficient LDL receptor-mediated cholesterol uptake	None	None	
	Glycogen storage disease type 1a (GSD1a)	*G6PC*		Differentiation to hepatocytes with elevated lipid and glycogen accumulation	None	None	
Haematological	Sickle cell anaemia	β-Globin alleles (β(s)/β(s)	OSKC Cre- excisable lentivirus	Genetically corrected hiPSCs generated using zinc finger nuclease homologous recombination	None	None	[111]
			OSKC piggyBac transposons	Heterozygous β(s)/β(A) gene correction in hiPSCs generated using zinc finger nuclease homologous recombination	None	None	[112]
	Fanconi anaemia (FA)	*Multifactorial*	OSKC retrovirus	Genetic correction of patient fibroblasts by lentiviral overexpression of FANCA or FANCD2 proteins, generation of hiPSCs and differentiation to phenotypically normal myeloid and erythroid hematopoietic progenitors	None	None	[113]
			OSKC retrovirus or multi-cistroniclentivirus	FA pathway complementation enables reprogramming of somatic cell to hiPSCs capable of hematopoietic differentiation	None	None	[114]
	Acquired myeloproliferativedisordes (MPDs)	*JAK2-V617F* somatic mutation in blood cells	OSKC retrovirus	Differentiation to CD34(+)CD45(+) hematopoietic progenitors with enhanced erythropoiesis and gene expression profiles similar to primary CD34(+) cells from the patient	None	None	[115]
	b-Thalassaemia major (Cooley's anaemia)	*ß-globin*	OSKC retrovirus	Genotyping	None	None	[116]
				Genetic correction of mutation by homologous recombination followed by implantation of hematopoietic progenitors into SCID mice to improve haemoglobin production	None	None	[117]
Eye	Retinitis pigmentosa (RP)	*RP1, RP9, PRPH2, RHO*	OSKC retrovirus	Rod photoreceptor cells recapitulated diseased phenotype of in vitro degeneration	α-Tocopherol (100 µM)	↑ Rhodopsin^+^ cells	[118]
					Ascorbic acid (200 µM)	No effect	
					β-Carotene (1.6 µM)	No effect	
	Gyrate atrophy (GA)	*OAT*	OSNLKC + SV40L Episomal	Gene-corrected hiPSCs generated	None	None	[119]
	Age-related cataract	*Multifactorial*	OSK lentivirus	hiPSCs differentiated to lens progenitor-like cells expressing lens-specific markers	None	None	[120]
Multi-organ	Down syndrome (DS)	*Trisomy 21*	OSKC retrovirus	Genotyping	None	None	[86]
	Shwachman-Bodian-Diamond syndrome (SBDS)	*SBDS*	OSKC retrovirus	Genotyping	None	None	[86]
	Dyskeratosiscongenita (DC)	*DKC1, TERC*	OSKC retrovirus	Disease model use to discovered novel mechanisms of telomerase regulation	None	None	[121]

O, OCT4; S, SOX2; K, KLF4; C, C-MYC; N, NANOG; L, LIN28; hiPSCs, human induced pluripotency stem cells; SMCs, smooth muscle cells; KD, knock-down; FPD, field potential duration; APD, action potential duration; BR, beat rate; EADs, early after-depolarisations; DADs, delayed after-depolarisations; N/S, not specified; N/A, not available.

Grey areas indicate where drug treatment has been tested.

Nevertheless, it is noteworthy that, with the exception of the eye disorder retinitis pigmentosa, only hiPSCs models affecting the heart and central nervous system have been used to evaluate effects of drug treatment in detail (Table 1; Fig. 1). This highlights several critical factors that are often overlooked in hiPSC technology: How will the phenotype of the disease be quantified in vitro? How will benefits of different methods of therapeutic intervention be evaluated? If a disease phenotype is present, how does it relate to the patient's condition? Is the therapy tested in vitro relevant to the patient, and is there potential for clinical translation? As shown in Table 1, the level of genetic and/or pharmacological characterisation in the majority (46/63) of hiPSC models is limited, and the answers to these questions are outstanding.

## Phenotype assessment in hiPSC-derived neurons and cardiomyocytes

Most progress has been made in phenotyping and evaluating drugs in hiPSC-based models of neurological and cardiac conditions (Table 1). Motor-, cortical- and dopaminergic-neurons from hiPSC harbouring mutations associated with neurodegenerative (e.g. Alzheimer's, Parkinson's and Huntington's diseases, schizophrenia) and neurodevelopmental disorders (e.g. Rett syndrome, spinal muscular atrophy, familial dysautonomia) have been successfully generated. Quantitative phenotyping of these cells has indicated severe defects in growth, migration and function compared to healthy controls. They therefore provide platforms for drug validation (Table 1). For example, the known anti-psychotic drug, loxapine, has been shown to improve neuronal connectivity in schizophrenia models [29], while compound E, a tobacco-derived γ-secretase inhibitor, decreased secretion of pathogenic Aβ42 in Alzheimer's models [30, 31]. Rett syndrome models have also been used for validation of experimental drugs such as gabazine, a GABA_A_ receptor antagonist [32].

Genetic disorders that affect the structure, ion channel composition and functionality in the heart also provide a quantifiable phenotypic readout. One of the consequences of the multi-system disorder of LEOPARD syndrome is cardiac hypertrophy, which has been partially phenocopied using hiPSC-cardiomyocytes [33]. The techniques of patch clamping and multi-electrode array (MEA) have proved valuable in interrogating electrophysiology from single or multi-cell clusters of cardiomyocytes, respectively [34]. Alterations in calcium handling can be visualised using realtime microscopy in the presence of calcium sensitive dyes [35]. Data from hiPSC lines carrying mutations that cause LQTS and catecholaminergic polymorphic ventricular tachycardia (CPVT) are starting to produce evidence that patient-relevant phenotypes and drug response can be recreated in vitro. In the case of LQTS2, caused by mutations in the *I*_Kr_ channel, hiPSC-derived cardiomyocytes developed arrhythmias when exposed to isoprenaline, a stressor used clinically to precipitate and diagnose the condition [34]. This effect could be reversed by applying the patient's own medication, nadolol, a β-blocker. Dantrolene and roscovitin, drugs known to be beneficial in moderating calcium flux, stabilised ion flux in hiPSC models of the calcium channel disorders, CPVT and Timothy syndrome (linked to LQT type 8), respectively 35–37.

Human induced pluripotent stem cell-cardiomyocytes are now providing novel routes to test more experimental drugs. The arrhythmias seen in the LQTS2 models were abolished by the potassium channel modulators, nicorandil and pinacidil (K^+^_ATP_ channel openers) or PD-118057 (*I*_Kr_ channel activator) [34, 38]. Encouragingly, it has been shown that hiPSC-cardiomyocytes can replicate relatively subtle differences between patients. hiPSCs were produced from a healthy donor as well as from a mother and daughter, wherein the mother was clinically asymptomatic (no arrhythmias) with a moderately prolonged QT interval and the daughter was symptomatic with an excessively prolonged QT interval (arrhythmias, syncope and seizure episodes). Recording action potential durations from the different hiPSC-cardiomyocytes showed that the clinical profile was reflected in vitro (i.e. action potential longest in the daughter's cells, then the mother's, then the healthy control) and only hiPSC-cardiomyocytes produced from the daughter developed spontaneous arrhythmias [34]. Establishing whether such in vitro to in vivo associations hold true for other conditions will be important for hiPSC technologies to become widely accepted.

## Assessing the need for humanised cardiotoxicity testing platforms

The ability to quantify functional responses in lineages such as hPSC-cardiomyocytes will likely find use in drug safety assessment. In recent years, high rates of drug attrition and withdrawal from market (because of unexpected cardiotoxicity) have imposed a multi-billion dollar burden on the pharmaceutical industry. More than ten drugs used to treat various non-cardiac conditions (e.g. inflammatory disease, psychosis, bacterial infection, pain) have been withdrawn from market because of unexpected side effects on the heart [39]. Side effects can damage the structural integrity and survival of cardiomyocytes, as is the case with the anti-inflammatory drug, Vioxx [39] and many anti-cancer drugs, such as doxorubicin [40]. Beat regularity and duration (QT prolongation or shortening) can also be affected, which can lead to polymorphic ventricular tachyarrhythmia, seizures and sudden death. Indeed, in 2010 this was the reason for the US FDA requesting withdrawal of propoxyphene, an opioid pain reliever marketed by Xanodyne Pharmaceuticals [41], and of sibutramine, a weight loss agent marketed by Abbott Laboratories [42]. With development costs of each drug averaging $1.5 billion, high profile withdrawals are extremely damaging for the companies involved, as well as for patients taking the medication; the serotonin agonist, cisparide, caused 125 deaths before its use ceased [43].

The use of suboptimal screening and safety assessment platforms underlies the reason for which drugs with potentially lethal side effects are not eliminated from the development pipeline before they reach the clinic. Early in most development pipelines, drugs are tested for channel modulating activity by utilising aneuploid cell lines (e.g. Chinese hamster ovary [CHO] or human embryonic kidney [HEK] cells) engineered to overexpress single ion channels. Such assays bear little relation to the complex multi-channel phenotype of functional cardiomyocytes [44]. This issue is illustrated by the in vitro culture responses seen with verapamil, a ‘safe’ drug in routine clinical use for treatment of hypertenstion, angina pectoris and cardiac arrhythmia. In CHO cells forced to overexpress *HERG*, verapamil blocks the potassium *I*_Kr_ channel, thereby predicting an association with prolonged QT interval [45]. In reality, while outward ion flux through *I*_Kr_ channels is blocked in functional cardiomyocytes, verapamil also blocks inward flux through L-type calcium channels (*I*_Ca-L_), and the overall effect on QT interval is cancelled out [45]. Similarly, ranolazine, a drug used to treat angina, blocks opposing sodium *I*_Na_ and potassium *I*_Kr_ channels, with limited effect on QT duration [46].

As discussed earlier, there are substantial differences in gene expression and physiology between species, which can limit the effectiveness of extrapolating toxicity from animals to humans. Indeed, data from non-rodents or rodents are respectively, 63 and 43% predictive of whether a drug will be toxic in humans. Even when data are combined from rodents (mice and rats) and non-rodents (dogs and monkeys), only 71% predictivity is achieved [47]. Notably, mice are at least 10× more tolerant to 37% of drugs than humans, while rats and dogs tolerate 4.5–100-fold the concentration of various chemotherapeutic agents as humans (e.g. ThioTEPA, Myleran, Actinomycin-D, Mitomycin C, Mithramycin, Fludarabine) [48]. Conversely, potentially valuable drugs might be eliminated during development because of overt toxicity in animals, when in fact they might be completely innocuous in humans. By way of example, chocolate and coffee can cause organ failure and death in dogs. This is because, relative to humans, the methylxanine ingredients, theobromine and caffeine, of these foods are poorly metabolised in dogs, which leads to potentially fatal toxic build up [49].

Despite these inadequacies, regulatory guidelines (e.g. international conference on harmonisation; ICH S7B) require extensive animal use in safety assessment because predictivity of current in vitro assays is insufficient. This has major implications for the number of animals used, and is not in line with the developing 3Rs (replacement, refinement and reduction of animal use) policies of many countries. For example, in the UK in 2008, a total of 475,290 animal procedures were performed to supply the needs of drug safety assessment and toxicity testing [50]. New EU regulation for the registration, evaluation, authorisation and restriction of chemicals (termed REACH) will require toxicological testing of 30,000 compounds, and some reports suggest that this will require up to 54 million animals over the next 10 years in Europe alone [50, 51].

These observations lead to the conclusion that any new human-based in vitro assays that improve or complement existing tests would benefit 1. patients through better drug safety; 2. the 3Rs, through reduced animal use; and 3. pharmaceutical companies, through reduced preclinical costs and drug withdrawals.

## Progress towards using hPSC-cardiomyocytes in cardiac safety assessment

In the last few years, tremendous progress has been made in improving the efficiency and robustness of cardiac differentiation from hPSCs, thereby providing a renewable source of human cardiomyocytes. The three differentiation strategies employed are formation of (i) three-dimensional aggregates known as embryoid bodies, (ii) two-dimensional monolayers or (iii) co-cultures with an inducer cell line such as END-2; these methods have recently been reviewed [11]. The cardiomyocytes display many of the gene expression patterns associated with in vivo development of the heart, including gene expression, ion channel formation, electrophysiological responsiveness and excitation-contraction coupling [52].

These attributes suggest that hPSC-cardiomyocytes could provide a human-based in vitro assay system for drug testing. Indeed, the pharmacological responses of hPSC-cardiomyocytes have been quantified from nearly 60 different compounds and drugs (Table 2). While the range of agents is extensive, most studies have only used one or two concentrations of drug that are at the upper end or exceed clinically relevant doses. Nonetheless, several important points are emerging, as considered below (see also Tables 1 and 2, and references therein).

**2:** Drug evaluation in hPSC-cardiomyocytes

AGENT	Mechanism of action	hPSC lines	Drug conc. (M)	Detection method	Obsrved effect on hPSC-CMs	Refs
2-APB	Cell permeate IP3R antagonist	hIH-I-clone 1&2; hfib2-5 (hiPSC)	2 µM	Laser confocal Ca^2+^ imaging	Significant decrease in whole-cell (Ca^2+^)_I_ transients amplitude and frequency	[38]
2,3-Butanedione monoxime	Uncompetitive ATPase inhibitor	H1 (hESC)	10^−3^ M	MEA	Arrested contraction	[122]
Acetylcholine	Muscarinic receptor agonist	SA002, SA121 (hESC)	10^−6^–10^−3^ M	Microscopy	↓ Beat rate	[123]
Adrenaline	β1-Adrenoceptor agonist	KhES1 (hESC), 201B7 (hiPSC)	0.5–50 µM	MEA	↑ Beat rate	[124]
		SA002, SA121 (hESC)	10^−9^–10^−5^	Microscopy		[123]
Atenolol	β1-Adrenoceptor antagonist	SA002, SA121 (hESC)	10^−8^–10^−6^	Microscopy	↓ Beat rate, blocked effect of adrenaline	
Amiodarone	K channel blocker	KhES1 (hESC), 201B7 (hiPSC)	1–100 µM	MEA	↓ Beat rate	[124]
Atropine	Competitive Ach inhibitor	SA002, SA121 (hESC)	10^−6^	Microscopy	Blocked effect of acetylcholine	[123]
ATX-II	*I*_Na,late_ enhancer	SA002 (hESC)	<1 µmol/L	Patch	No effect on APD and triangulation	[61]
BaCl_2_	*I*_K1_ blocker	SA002 (hESC)	10 µM	Patch	No effect on triangulation or AP prolongation	
		H1 (hESC)	0.5 mM		Increased the slope of diastolic depolarisation	[63]
Bay K8644	Calcium channel enhancer	SA002 (hESC)	1 µM	Patch	APD_50_ and APD_90_ increased by 27%; no effect on triangulation	[61]
		hiPSC (iCells, Cellular Dynamics International)	10 and 100 nM		No or little stimulation of Ca channel current amplitude. 100 nM, inhibited current. Slowed Ca channel inactivation/activation	[125]
Caffeine	Inducer of SR Ca^2+^ release	H1, HES2 (hESC)	10 mM	Fura-2/AM	↑ Cytosolic Ca	[126]
		hiPSC, H9.2 (hESC)	10 mM	MEA	Minor increase in diastolic [Ca2^+^]_i_ ratio	[127]
		hIH-I-clone 1&2; hfib2-5 (hiPSC)	20 mM	Laser confocal Ca^2+^ imaging	Increase in Ca induced transient amplitude-dose dependent increase	[38]
Carbamylcholine	Muscarinic receptor agonist	hFib2-iPS (hiPSC)	1 and 10 µM	MEA	Dose-dependent ↓ in beat rate	[128]
		H2 (hESC)	0.1 mM	Patch	↓ Beat rate	[129]
		H7 (hESC)	10 µM	Patch	Significant drop in beat rate	[130]
		H9.2 (hESC)	1 µM	MEA	↓ In beat rate	[131]
CGP 20712A	β1-Adrenoceptor antagonist	H7 (hESC)	0.3 µM	Patch	Reduced beating rate and further increased in conjunction with isoprenaline. No significant effect on relaxation (R_50_ & R_90_)	[130]
Chromanol 293B	*I*_Ks_ block	hFib2-iPS (hiPSC)	10 and 30 µM	MEA	Dose dependent ↑ in cFPD	[128]
		SA002 (hESC)	100 µM	Patch	Prolonged APD_90_; no EAD; no effect on triangulation	[61]
		201B7 (hiPSC)	N/S	Patch	Time and dose dependent AP prolongation	[132]
Cisapride	Serotonin 5HT agonist	UTA.00514.LQT2 (hiPSC)	40–330 nM	MEA	No ↑ in arrhythmogenicity	[82]
		LQT2-hiPSC	100 nM	MEA	↑ cFPD, ↑ arrythmogenicity	[38]
		HES2, HES3 (hESC)	0.1 nM–1 µM	MEA	↑ FPD only at higher concentrations	[54]
		SA002 (hESC)	0.01–1 µM	Patch	Increase in APD_90_; triangulation increased and 1/11 clusters showed EAD at 1 µmol/L	[61]
Clenbuterol	β2-Adrenoceptor agonist	H1, H7 and H9 (hESC) and H9.1 and H9.2 (clonal)	10^−7^–10^−9^ M	Patch	No response to contractions at day 22 and 39 of differentiations. At day 61 and 72 increase in beating frequency	[133]
Diltiazem	L-type Ca^2+^ channel blocker	H9.2 (hESC)	1–10 µM	MEA/patch	No effect on conduction or automaticity	[134]
		201B7 (hiPSC)	0.01 and 1 µM	Patch	Shortened APD_30_ and APD_90_; no affect on APD_30-90_	[132]
		H1, H7 and H9 (hESC) and H9.1 and H9.2 (clonal)	10^−7^–10^−5^ M	Patch	Dose dependent ↓ in beating frequency. At 10^−7^ mol/L frequency was significantly reduced and stopped beating at 10^−5^ mol/L	[133]
Digoxin	Inhibit Na^+^/K^+^-ATPase	hiPSC (iCells, Cellular Dynamics)	0.3–10 µM	MEA	At 3 µM, reduced Na^+^-spike amplitude, shortened FPDcf and increased Ca^2+^-wave amplitude	[135]
Domperidone	Multiple channel blocker	HES2, HES3 (hESC)	0.1 nM–100 µM	MEA	Minor ↑ in FPD at ETPC unbound (5–19 nM), biphasic dose-dependent ↑ in FPD at higher concentrations	[54]
E4031	*I*_Kr_ blocker	UTA.00514.LQT2 (hiPSC)	500 nM	MEA	↑ In arrhythmogenicity (effect greater in diseased lines)	[82]
		hiPSC	3–100 nM	Patch	↑ APD50, ↑ APD90 and AP triangulation	[79]
		LQT2-hiPSC	500 nM	MEA/patch	↑ APD/cFPD, ↑ arrythmogenicity and development of EADs	[38]
		LQT2-hiPSC	10^−1^–10^−3^ M	MEA/patch	↑ cFPD/APD (77% ↑ in patient CMs as opposed to 50% in control CMs); EADs in 30% of LQT2-CMs vs. none in controls	[34]
		SA002 (hESC)	0.03–1 µM	Patch	Dose-dependent ↑ APD90, ↑ AP triangulation, EADs at high concentrations	[136]
		hESC	100 nM	Patch	Prolongation of AP; greater effect on APD90 than APD 50	[137]
		HES2, HES3 (hESC)	30–300 nM	MEA	Dose dependent ↑ in FPD, ↓ in beat rate at micromolar concentrations, EADs between 1^−3^ µM in ¾ experiments	[54]
		201B7 (hiPSC)	10–100 nM	MEA	↑ FPD	[138]
			0.01, 0.1 and 1 µM	Patch	Prolonged APD_30_, APD_90_ and APD_30-90_ in concentration dependent manner; EAD in 2/4 cells	[132]
		hFib2-iPS (hiPSC)	500 and 1,000 nM	MEA	Dose dependent ↑ in cFPD	[128]
		H1 (hESC)	10 µM	Patch	Non-reversible ↑ APD after 30 seconds. Late stage differentiation depolarised diastolic potential/↑ frequency of spontaneous AP	[63]
			500 nM	Patch	AP ↑ in both atrial and ventricular like-CMs but APD 90 and APD50 response dependent on subtype	[139]
Erythromycin	*I*_Kr_ blocker	UTA.00514.LQT2 (hiPSC)	1.5–16 µM	MEA	No ↑ in arrhythmogenicity	[82]
Flecainide	Na channel blocker	KhES1 (hESC), 201B7 (hiPSC)	0.1–10 µM	MEA	No effect on beat rate	[124]
Forskolin	Adenylatecyclase stimulator	H9.2 (hESC)	1 µM	MEA	↑ beat rate	[131]
		SA002, SA121 (hESC)	10^−12^–10^−7^ M	Microscopy	Increase in beat rate	[123]
FPL 64176	L-type Ca^2+^ channel activator	hiPSC (iCells, Cellular Dynamics); hESC (Geron)	100–1,000 nM	Patch	Variable ↑ in Ca channel current amplitude. Slowed Ca channel activation, inactivation and tail current kinetics	[125]
Heptanol	Gap junction blocker	H1 (hESC)	0.4 mM	MEA	uncoupling of cardiomyocytes	[122]
IBMX (Isobutyl methylxanthine)		H9.2 (hESC)	10 µM	MEA	↑ beat rate	[131]
	Phosphodiesterase inhibitor	H1, H7 and H9 (hESC) and H9.1 and H9.2 (clonal)		patch	Dose dependent increase in contraction rate	[133]
ICI 118,551	β2-Adrenoceptor antagonist	H7 (hESC)	50 nM	Patch	In presence of ICI, increase in beating rate with isoprenaline reduced. Significant acceleration of relaxation (R_90_)	[130]
Isoprenaline	β1/β2-Adrenoceptor agonist	UTA.00514.LQT2 (hiPSC)	80 nM	MEA	↑ Chronotropy (both diseased and control lines)	[82]
		H7 (hESC)	0.1 µM	Patch	Increase in beat rate; R50 and R90, were reduced	[130]
			0.001–10 µM		Dose dependent increase in beat rate; EC_50_ of 12.9 nM	
		LQT2 hiPSC	10^−1^–10^−3^	MEA/patch	↓ in cFPD, APD, APD50 and APD90 (patient lines significantly more sensitive); EADs in 25% of patient, but none of control CMs	[34]
		IMR90 C1, IMR90 C4 (hiPSC). H1, H9 (hESC)	1 µM	Patch	↓ In APD, ↑ in beat rate	[140]
		HUES7, NOTT1 (hESC)	1 µM	MEA	↑ Beat rate, ↓ FPD	[8]
		H2 (hESC)	1 µM	Patch	↑ Beat rate	[129]
		iPSC, H9.2hESC(hESC)	10^−9^–10^−7^ M	MEA	Concentration dependent positive inotropiceffect	[127]
		CBiPSC6.2 (hiPSC)	20 µM	Optical voltage maps	↓ AP, ↑ conduction velocity	[141]
		SA002 (hESC)	0.1 µM	Patch	↑ Beating frequency, ↓ APD; suppresses E4031-induced EADs	[136]
		hFib2-iPS (hiPSC)	1 and 10 µM	MEA	Dose-dependent ↑ in beat rate	[128]
		LQT1-hiPSC	100 nM	Patch	15% ↑ in APD90/AP, ↑ risk of arrhythmias, EADs	[81]
		LQT2-hiPSC	10 µM	MEA	↑ Chronotropy	[38]
		KhES1 (hESC), 201B7 (hiPSC)	0.01–1 µM	MEA	Dose-dependent ↑ in beat rate	[124]
		201B7 (hiPSC)	200–500 nM	MEA	↑ Beat rate, ↓ FPD	[138]
		H1 (hESC)	1 µM	MEA	↑ Beating frequency	[122]
		H9.2 (hESC)	1 µM	MEA	↑ Beat rate	[131]
		H1, H7 and H9 (hESC) and H9.1 and H9.2 (clonal)	10^−5^–10^−9^ M	Patch	Enhanced the contraction rate in dose dependent manner, at differentiation day 15–20	[133]
		H1, H7, H9, H14 (hESC)	1 µmol/L	Patch	Increase in magnitude of contraction	[139]
Ketoconazole	Cyp34a inhibitor	HES2, HES3 (hESC)	0.3 nM–30 µM	MEA	No effect on FPD	[54]
Lacidipine	L-type Ca^2+^ channel blocker	H1 (hESC)	10 µM	Patch	Reduction in plateau duration and height of AP profile recorded from 40 day old beating cluster	[63]
Lidocaine	Voltage-gated Na^+^ channel inhibitor	HES2, HES3 (hESC)	0 pM–100 µM	MEA	Cessation of beating in the 30–100 µM range	[54]
		H1 (hESC)	100 µM	MEA	↓ Conduction rate	[122]
		201B7 (hiPSC)	100, 1,000 µM	Patch	Concentration dependent inhibition of *I*_Na_	[131]
Mexiletine	Na^+^ channel blocker	KhES1 (hESC), 201B7 (hiPSC)	0.1–10 µM	MEA	No effect on beat rate	[124]
Nadolol	β-Adrenoceptor antagonist	LQT2 hiPSC	10^−1^–10^−3^ M	Patch	Attenuation of isoprenaline-induced arrythmias	[34]
Nicorandil	*I*_KATP_ opener	LQT2 hiPSC	10^−1^–10^−3^ M	Patch	↓ APD, abolishment of spontaneously occurring EADs	[34]
Nifedipine	L-type Ca^2+^ channel blocker	hiPSC	3–100 nM	Patch	↓ APD10, ↓ APD50, ↓ APD90	[79]
		LQT2-hiPSC	1 µM	MEA/patch	↓ cFPD, ↓ APD and ↓ APD90; eliminated EADs and triggered beats	[38]
		HES2, HES3 (hESC)	10 nM–1 µM	MEA	Dose dependent ↓ in FPD, ↑ in beat rate, but no arrhythmic activity, loss of spontaneous activity between 300 nM and 1 µM	[54]
		H9.2 (hESC)	0.1–1 µM	MEA/patch	No effect on conduction or automaticity	[134]
		SA002 (hESC)	10 nM	Patch	Shortened AP; negated effect of BAY K8644	[61]
		hiPSC (iCells, Cellular Dynamics)	0.01–3 µM	MEA	Accelerated beat rate; shortened FDPcf; reduced Ca wave amplitude; reduction of Na spike amplitude by 20% at 3 µM	[135]
		hiPSC (iCells, Cellular Dynamics); hESC(Geron)	6 nM(hESc); 3 nM(hiPSC)	Patch	Inhibit Ca^2+^ channel currents	[125]
		hIH-I-clone 1&2; hfib2-5 (hiPSC)	1 µM	Laser confocal Ca^2+^ imaging	Elimination of whole cell (Ca^2+^)_I_ transients; decrease in (Ca^2+^)_I_ transients amplitude at lower nifedipine concentration	[142]
Ouabain	Inhibit Na^+^/K^+^-ATPase	hiPSC (iCells, Cellular Dynamics)	0.3–10 µM	MEA	Time and dose dependent-reduced Na^+^-spike amplitude, shortened FPDcf and increased Ca^2+^-wave amplitude	[135]
PD-118057	Type 2 *I*_Kr_ channel enhancer	LQT2 hiPSC	10^−1^–10^−3^ M	Patch	↓ APD	[34]
Phenoxybenzamine	α1-/α2-Adrenoceptor antagonist	SA 002 and SA 121 (hESC)	10^−7^–10^−5^ M	Microscopy	Reduces beat rate	[123]
Phenylephrine	α1-Adrenoceptor antagonist	HES2 (hESC)	0.1 mM	Patch	↑ Beat rate	[129]
		H1, H7 and H9 (hESC) and H9.1 and H9.2 (clonal)	10^−4^–10^−8^ M	Patch	↑ Contraction rate in dose dependent manner, at differentiation day 15–20	[133]
		SA 002 and SA 121 (hESC)	10^−7^–10^−11^ M	Patch	Dose dependent increase in contractile activity	[123]
Pinacidil	*I*_KATP_ opener	CBiPSC6.2 (hiPSC)	100 µM	Optical voltage maps	↓ AP, ↑ conduction velocity	[141]
		LQT2-hiPSC	1 µM	MEA/patch	↓ cFPD, ↓ APD and ↓ APD90, eliminated EADs/triggered beats	[38]
Procainamide	Na^+^ channel blocker	KhES1 (hESC), 201B7 (hiPSC)	10–1,000 µM	MEA	No effect on beat rate	[124]
Propranolol	β-Adrenoceptor antagonist	LQT1-hiPSC	200 nM	Patch	Attenuation of catecholamine-induced tachyarrhythmias	[81]
		LQT2 hiPSC	10^−1^–10^−3^ M	MEA/patch	Attenuation of isoprenaline-induced arrhythmias	[34]
		KhES1 (hESC), 201B7 (hiPSC)	0.3–30 µM	MEA	No effect on beat rate, blocked effect of isoprenaline	[123]
Quinidine	Multiple ion channel blocker (*I*_to_, *I*_Katp_, *I*_KI_, *I*_Kr_, *I*_Ks_, *I*_Ca_, *I*_NaL_)	hFib2-iPS (hiPSC)	100 µM	MEA	↑ In cFPD, variable effect on the amplitude of the 1st negative peak of the FP, variable effect on chronotropy	[128]
		HES2, HES3 (hESC)	0.1 nM–100 µM	MEA	Dose dependent ↑ in FPD and QTi (i.e. prolonged FPD at physiologically relevant plasma concentrations)	[54]
		201B7 (hiPSC)	4–50 µM	MEA	↓ FP amplitude	[138]
		HES2 (hESC)	1 µM	MEA	↑ APD	[143]
Ranolazine	Multiple ion channel blocker (*I*_Kr_, *I*_Ca_, *I*_NaL_)	LQT2-hiPSC	15–50 µM	MEA/patch	No change in cFPD/APD, pronounced anti-arrythmic effect	[38]
Ryanodine	Ryanodine receptor inhibitor	H1, HES2 (hESC)	10 µM	Fura-2/MEA	↓ Ca current amplitude	[126]
		hIH-I-clone 1&2; hfib2-5 (hiPSC)	10 µM	Laser confocal Ca^2+^ imaging	Significant reduction in Ca2+ release. Increasing doses of ryanodine led to increase in % decrease in (Ca^2+^)_I_	[142]
		H9.2 (hESC)	10 µM	Fura-2/MEA	No effect on contraction	[144]
		hiPSC, H9.2 (hESC)	10 µM	MEA	↓ In contraction in iPSC-CMs, No effect on contractionsinhESC-CMs	[127]
Sertindole	Multiple ion channel blocker (*I*_Kr_, *I*_Ca_, *I*_NaL_)	HES2, HES3 (hESC)	0.01 nM–100 µM	MEA	No effect on FPD at ETPC unbound (0.02–1.59 nM), relatively weak ↑ in FPD at higher concentrations	[54]
Sotalol	*I*_Kr_ blocker	UTA.00514.LQT2hiPSC	19 µM	MEA	↑ In arrhythmogenicity (only in diseased lines)	[82]
		HES2, HES3 (hESC)	0.1 nM–100 µM	MEA	Dose dependent ↑ in FPD and QTi (i.e. prolong FPD at physiologically relevant plasma concentrations)	[54]
		H1 (hESC)	300 µM	MEA	↑ FP duration; time dependent ↑ of repolarisation phase; no significant change in beating rate	[145]
Sparfloxacin	*I*_Kr_ blocker	HES2, HES3 (hESC)	0.1 nM–100 µM	MEA	No effect on FPD at ETPC unbound (0.19–1.76 µM), ↑ FPD at higher concentrations	[54]
Sunitinib malate	*I*_Kr_ blocker	iCells, Cellular Dynamics	1–30 µM	MEA	↑ cFPD, dose-dependent ↓ in beat rate, arrhythmic beats at 10 µM, with altered amplitude and beat duration at 30 µM	[146]
Tetrodotoxin	Voltage-gated Na^+^ channel inhibitor	hiPSC	3–30 µM	Patch	Delay in upstroke, ↓ d*V*/d*t*_max_	[79]
		hFib2-iPS (hiPSC)	10 µM	MEA	↓ In conduction time	[128]
		H9.2 (hESC)	10–100 µM	MEA	↓ Conduction rate and beat rate, local conduction blocks	[134]
		Miz-hES2 and HSF-6 (hESC)	200 nM	Patch	Complete depletion of action potential	[147]
Terfenadine	Multiple ion channel blocker (*I*_Kr_, *I*_Ca_, *I*_NaL_)	HES2, HES3 (hESC)	0.1 nM–100 µM	MEA	No effect on FPD at ETPC unbound (0.1–0.29 nM), ↑ FPD at higher concentrations but ↓ FPD at micromolar concentrations	[54]
			0.01, 0.1 and 1 µM	Patch	Prolonged APD_30_, APD_90_ and APD_30-90_	[132]
Thapsigargin	SERCA2A inhibitor	H1, HES2 (hESC)	0.1–1 µM	Fura-2/AM	↓ Amplitude of Ca transients	[126]
		H9.2 (hESC)	10 nM	Fura-2/MEA	No effect on contraction	[144]
U73122	Phospholipase C inhibitor	hIH-I-clone 1&2; hfib2-5 (hiPSC)	2 µM	Confocal Ca^2+^ imaging	Significant ↓ in Ca^2+^ release. Increasing doses of ryanodine led to increase in % decrease in (Ca^2+^)_I_	[142]
Verapamil	Multiple ion channel blocker (*I*_Kr_, *I*_Ca_)	hFib2-iPS (hiPSC)	1 and 5 µM	MEA	Dose dependent ↓ in cFPD and beating frequency (complete arrest of spontaneous beating frequency at 5 µmol/L	[128]
		hIH-I-clone 1&2; hfib2-5 (hiPSC)	10 µM	Confocal Ca^2+^ imaging	Dose dependent ↓ in whole cell (Ca^2+^)_I_ transients amplitude in hIH-I and hfib2-5	[142]
		KhES1 (hESC), 201B7 (hiPSC)	0.1–10 µM	MEA	Dose-dependent ↓ in beat rate	[124]
		HES2, HES3 (hESC)	25–81 nM	MEA	Minor FPD shortening at ETPC unbound (25–81 nM), greater ↓ in FPD at higher concentrations	[152]
		201B7 (hiPSC)	10–1,000 nM	MEA	↓ FPD	[138]
			0.01, 0.1 and 1 µM	Patch	Shortening of APD_30_, APD_90_; prolongation of APD_30-90_	[132]
		HES2 (hESC)	5 µM	Fura-2/patch	↓ Beat rate	[129]
		SA002, SA121 (hESC)	10^−12^–10^−9^ M	Microscopy	Reduced or stopped contractile activity	[123]
Veratridine	Na channel modulator	hESC	10 mM	Patch	Prolonged AP/increased triangulation; reversible	[137]
Zatebradine	*I*_Kr_ blocker	SA002 (hESC)	0.1, 1 and 10 µM	Patch	Increasing concentration caused slowing of beating and changes APD and triangulation. EADs	[61]
		H1 (hESC)	10 µM	Patch	↓ Depolarisation rate and spontaneous rhythm	[63]
ZD7288	*I*_f_ blocker	H1 (hESC)	NA	MEA	↓ Beating frequency	[122]

hPSC, human pluripotent stem cells; hESC, human embryonic stem cells; hiPSC, human induced pluripotent stem cells; N/S, not specified; patch, patch clamp electrophysiology; MEA, multi-electrode array; APD, action potential duration; FPD, field potential duration; EADs, early after depolarisations; QTi, QT interval; CM, cardiomyocytes; ETPC, estimated unbound therapeutic plasma concentrations.

First, functionality in hPSC-cardiomyocytes has been shown for many of the key ion channels (potassium: *I*_Ks_, *I*_Kr_, *I*_f_, *I*_to_, *I*_K1_; sodium: *I*_Na_; calcium: *I*_Ca-L_, SERCA2a) and regulator molecules (e.g. receptors: muscarinic, adrenoceptors, acetylcholine, ryanodine) found at the cell membrane or in the sarcoplasmic reticulum. Second, functional responses can be quantified by methods of relevance to the pharmaceutical industry, such as patch clamp electrophysiology and calcium detection. Third, responses can be measured from cardiomyocytes derived from a range of healthy and disease-carrying hPSC lines. Fourth, the complex multi-ion channel phenotype of hPSC-cardiomyocytes provides an advantage over CHO cells forced to overexpress a single channel. Dual channel blocking agents such verapamil (blocks *I*_Kr_ and *I*_Ca-L_) and ranolazine (blocks *I*_Kr_ and *I*_Na_) are QT-neutral when clinically relevant doses are applied to hPSC-cardiomyocytes. Fifth, in some cases, hPSC-cardiomyocytes can detect toxic effects at lower doses than is possible in animal systems. We have found that the *I*_Kr_ blocker, risperidone, causes increased field potential duration of hPSC-cardiomyocytes at 0.1 µM [46], but data from GlaxoSmithKline indicate that prolongation occurs in guinea-pig myocytes at 1 µM. Moreover, direct comparison between hPSC-cardiomyocytes and myocytes isolated from dogs or rabbits concluded that the human cells more accurately predicted moxifloxacin-induced cardiotoxicity [53]. Finally, a careful study examined drug effects over a 6-log dose-response range that covered the estimated unbound therapeutic plasma concentrations [54]. There was good association between clinical and hPSC-cardiomyocyte toxicity for drugs such as quinidine and d,l-sotalol known to prolong QT interval, whereas drugs with a low incidence of arrhythmogenesis (e.g. cisapride, terfenadine, sertindole, sparfloxacin) only caused prolongation of field potential duration at higher doses [54].

## Limitations and challenges to overcome in hPSC technology

The emerging data for disease modelling and drug screening are encouraging. However, this is a new field with limitations yet to be overcome. Although hESCs are often considered the gold standard, these cells are derived from spare embryos donated by couples experiencing fertility problems, hence the need for in vitro fertilisation (IVF) treatment. It is known that different methods of embryo culture can alter epigenetic status [55]. For hiPSC derivation, delivery of reprogramming factors can be achieved by viral (e.g. retroviruses, lentiviruses, adenoviruses, sendaivirus) or non-viral (episomes, plasmids, miRNA, mRNA and protein) strategies [56]. It is notable that virtually all disease models have used the ‘original’ retroviral and lentiviral methods (Table 1) [2, 3], and a potential concern is random integration of the viral genome into the host genome [57]. Assessment is further complicated, because it depends on whether the reprogramming factors are contained on single or multiple vectors, and whether small molecule enhancers of hiPSC production were used [56, 58]. There is not yet a consensus on the cell type to reprogram [56], although skin and blood cells are preferred because of the ease of patient consent, minimal discomfort to the patient, and accessibility. Each of these variables has the capacity to alter the genotype, epigenome and phenotype of the hiPSCs produced, as well as the subsequently derived differentiated lineages. Therefore, it is difficult to know whether problems reported for hiPSC (e.g. transfer of epigenetic legacy from somatic cells to hiPSC, improper reprogramming/disease modelling [e.g. Fragile X] or genetic instability) [59] are inherent to the technology or are a consequence of the reprogramming method(s) used. Detailed studies to resolve these issues are required, as is a consensus of the best cell type to reprogram and how.

In addition to the careful consideration of how disease presentation will be phenotyped in vitro (discussed earlier), there is also an issue of whether hPSC derivatives mature sufficiently in culture to make them fit for their intended purpose. To date, drug treatment and phenotypic studies in hiPSC-derived neurons have been more successful for neurodevelopmental disorders than late-onset neurodegenerative disorders, likely because of the foetal-like properties of the cells [60]. The absence of functional potassium channels (*I*_K1_) and shifted activation of sodium channels (*I*_Na_) indicates an immature status of hPSC-cardiomyocytes, and has raised concerns about their suitability in drug screening [61]. Therefore it is encouraging that maturation of hPSC-cardiomyocytes can be facilitated by prolonged time culture [62, 63], transgenic overexpression of calsequestrin [64], formation of 3D aggregates [62], tissue-engineered constructs and mechanical stress [65, 66].

It is unlikely that hiPSC technology will successfully model all disorders. The epigenetic status that underlies some diseases will be erased during somatic cell reprogramming, while for other conditions a suitable phenotype may not be present in an in vitro setting [59]. Although several studies have now demonstrated robust association with the phenotypes and drug responses seen in hiPSCs models with known patient pathologies (e.g. LQTS), similar validation is required for a broad range of conditions (Table 1). The timing of some late onset conditions may exceed the lifespan of hiPSC-derivatives in culture, and innovative strategies are required. For example, the dopaminergic neurons differentiated from hiPSCs carrying a mutation in the PINK1 gene (causes Parkinson's disease) only showed altered patterns of survival when additionally treated with a mitochondrial stressor [67]. Finally, differentiation of the hiPSC into relevant cell types is necessary. So far, hiPSC modelling has been restricted to about 10 tissue or organ systems (Table 1) and future work will be needed to expand this range.

## Industrial scalability of hPSC technologies

For hPSC derivatives to be used for disease modelling and drug screening at an industrial level (Fig. 1), sufficient numbers of cells need to be produced in a cost-effective manner. Undifferentiated hPSCs have been produced using stirred bioreactors in suspension [68] and using fully automated robotic platforms such as the CompacT SelecT, which cultures adherent cells in up to 90 T175 flasks [69]. However, the cost of the reagents for hPSC culture is prohibitive because of the reliance of expensive culture media that contain various growth factors. To this end, high throughput screening has sought to identify putative chemicals that maintain pluripotency in the absence of growth factors or that improve cell survival after passage 70–72. Such approaches have identified a series of inhibitors of the Rho kinase pathway and prosurvival compounds such as Y27632 that are now used by many labs during routine hPSC culture. The same degree of success has not been achieved in replacing basic fibroblast growth factor (bFGF), which remains the gold standard for maintaining hPSC pluripotency in many labs.

Similar to the undifferentiated state, scaled production of differentiated lineages has been achieved, but also tends to rely on costly growth factors; in the case of hPSC-cardiomyocytes these typically include bFGF, bone morphogenetic protein (BMP4) and activin A [11]. Commercial production of hPSC-cardiomyocytes is now in progress, with GE-healthcare, Cellular Dynamics International and Cellartis/Cellectis charging approximately $2000–3000 per vial of ∼1 million cells. It is encouraging that small molecules that promote cardiac differentiation are being identified from high throughput screens and from rational compound selection (Table 3). Time- and concentration-dependent application of the BMP inhibitor, dorsomorphin, has proved to be highly effective in improving cardiomyocyte differentiation efficiencies [73]. In time, it is hoped that such strategies will allow hPSC-cardiomyocytes to be produced to short time scales, in large quantities at low cost. This goal has been achieved for production of >3 × 10^9^ mPSC-cardiomyocytes in stirred bioreactors [74]. Elegant work has also shown pipeline conversion of mouse fibroblasts into iPSCs and then into iPSC-cardiomyocytes in a single suspension bioreactor [75]; the challenge now is to translate the high efficiency ‘inducible secondary’ iPSC reprogramming into a technology that is compatible with human cells.

**Table 3:** Agents that influence cardiomyocyte differentiation of human pluripotent stem cells

	Agent	Cells	When added	Conc.	Observations	Refs
Small molecules	Ascorbic acid	hiPSC	Throughout differentiation	50 µg/mL	Improved cardiac differentiation and maturation	[148]
	5′-Azacytidine	H9 hESC	Day 6–8 of differentiation	1 or 10 µM	Increased aMHC expression	[133]
	DMSO	HUES7, HUES9	EBs in suspension and 24–48 hours postplating	0.01%	Upregulation of mesoderm markers	[149]
	Retinoic acid	H9 hESC	Postplating of EBs	1 µM	Activate ectodermal and mesodermal markers	[150]
	ITS	hESC, hiPSC	Day 0–2 and 4 onwards	1×	Insulin from d2–d4 inhibited cardiac specification	[141]
	Cyclosporin-A	hiPSC	d8 of END2 co-culture (hiPSC)	3 µg/mL	Number of beating colonies increased	[151]
Inhibitors	SB203580 (p38 MAPK inhibitor)	H9 hESC	Day 4–6 of EB differentiation	5–10 µM	2.1-fold increase in cardiomyocytes	[152]
		HES2, 3, 4 hESC	Day 0 of EB differentiation	10 µM	One-time addition increased percentage of beating EBs	[153]
	SB431542 (inhibitor of TGF-β/Nodal/Activin pathway)	hESC, hiPSC	Day 3–5 of EB differentiation	5.4 µM	aMHC RNA increased by 70%	[73]
	IWP-4(Wnt inhibitor)	HES3, H9, MEL1 hESC	Day 3–15 monolayer differentiation	5 µM	IWP-4 induced expression of cardiac markers	[154]
	IWP-3 (Wnt inhibitor)	hESC	Day 4–5 on plating of EBs	2 µM	Promoted cardiogenesis by about 40 times compared to DKK1	[155]
	IWR-1(Wnt inhibitor)	hESC	Day 4–5 on plating of EBs	4 µM	Maximal cardiac induction by IWR-1 corresponds from day 4–5	
	53AH (analogue of IWR-1)	hESC	Day 4–5 on plating of EBs	1 µM	Promoted cardiogenesis by about 40-fold compared to DKK1	
	XAV939 (inhibitor of tankyrase)	hESC	Day 4–5 on plating of EBs	2.5 µM	Promoted cardiogenesis by about 40-fold compared to DKK1	
	DKK1 (Wnt inhibitor)	H7, H1 hESC	Day 5–11 monolayer differentiation	200 ng/mL	Increased cardiomyocyte generation	[156]
	SU5402 (FGF receptor inhibitor)	hESC, hiPSC	4 or 6 days in culture	1 µM	Synergy between BMP2, Wnt3a and SU5402 (FGF receptor inhibitor) facilitate precardiac mesoderm	[157]
	Noggin (BMP4 inhibitor)	H7 hESC	Day 4–5 in differentiation media	250 ng/mL	Timed inhibition increased cardiac differentiation efficiency	[158]
	Dorsomorphin (BMP inhibitor)	hESC, hiPSC	Day 3–5 of EB differentiation	0.25 µM	In presence of SB431542 and dorsomorphin, cTnT positive cells increased fourfold	[73]
	BMS-189453 (RA receptor antagonist)	H7 hESC	Day 6–9 in differentiation media	1 µM	Timed inhibition of RA signalling promotes cardiac differentiation	[158]
Growth factors	WNT3a	HUES1, 7, 8 hESC	Day 1–4 of differentiation	25 ng/mL	Wnt3a and BMP4 are prominent cytokines in the posterior primitive streak and direct cells toward mesoderm	[159]
	TGFbeta1	H7 hESC	Pre-differentiation culture	0.5 ng/mL	Used in culture and pre-treatment of undifferentiated hPSCs	[160]
	FGF-2	hESC, hiPSC	Day 0–2 of EB differentiation	5 ng/mL	Combination of BMP4 and FGF2 was determined to be necessary for efficient cardiac differentiation	[141]
	EGF	H9 hESC	Postplating of EBs	100 ng/mL	Factors (EGF,RA,BMP4 and bFGF) activate ectodermal and mesodermal markers	[150]
	Activin-A	HES3, H9, MEL1 hESC	Day 0–3 of differentiation	6 ng/mL	Cardiomyocyte induction in RPMI/B27 media supplemented with activin A and BMP4	[154]
	BMP4	hESC, hiPSC	Day 0–2 of EB differentiation	25 ng/mL	Combination of BMP4 and FGF2 was determined to be necessary for efficient cardiac differentiation	[141]
		H1 hESC	4 Days in EB suspension	25 ng/mL	BMP4 treatment promotes cardiac induction from hESCs	[161]
	BMP2	hESC, hiPSC	4 or 6 days in culture	10 ng/mL	Synergy between BMP2, Wnt3a and SU5402 (FGF receptor inhibitor) facilitate precardiac mesoderm	[157]

DMSO, dimethyl sulphoxide; ITS, insulin-transferrin-selenium; IWP, inhibitor of WNT production; DKK1, Dickkopf-related protein 1; EGF, epidermal growth factor; WNT, wingless-int; BMP, bone morphogenetic protein; RA, retinoic acid; FGF, fibroblast growth factor; TGF-beta, transforming growth factor beta; cTnT, cardiac troponin-T; EBs, embryoid bodies; hiPSCs, human induced pluripotent stem cells; hESCs, human embryonic stem cells; aMHC, alpha myosin heavy chain.

## Progress towards high throughput analysis

In an industrial setting, drug discovery and safety evaluation relies on high content imaging of many thousands of wells in 96-, 384- and 1,536-well plates (Fig. 1). Various manufacturers offer fully automated platforms [76] such as BD pathway (BD Biosciences), In Cell Analyser 2000 (GE-healthcare), ImageXpress (Molecular Devices), Opera (Perkin Elmer) and Cellomics Arrayscan (ThermoFisher). These deliver a vast array of information on cell physiology and function, including cell number, cell shape/size, proliferation, viability, membrane integrity, phagocytosis, apoptosis, cell migration, cell-cell contacts and organelle health (e.g. numbers, size, shape, activity of nucleus, mitochondria, lysosomes) [77]. Fluorescent assays are also used to readout on G-protein coupled receptor (GPCR) activity, calcium handling and transgenic reporter expression [77]. As discussed above, such platforms have been used to evaluate molecules that help maintain pluripotency or promote differentiation of hPSCs but they are starting to find use in phenotypic evaluation of differentiated cells. The Cellomics Arrayscan platform was used to evaluate the effect of various modulators of hypertrophy (e.g. angiotensin II, phenylephrine, p38-MAPK) on cell morphology of hPSC-cardiomyocytes by examining 1,000–1,500 cells per well in 96-well plate formats [78]. Data have been presented by Cellular Dynamics International on quantification of the cardiotoxic effect of valinomycin, etoposide and rotenone in hPSC-cardiomyocytes using high content imaging of changes in mitochondrial and lysosomal physiology, DNA damage and oxidative stress. At a recent Predictive Toxicology Meeting in London (February 2012), data from GE-healthcare showed how 26 anti-cancer agents changed 19 different cell morphological and functional parameters in hPSC-cardiomyocytes. The analysis was carried out on three replicates, two timepoints and seven doses in a 384-well plate format using the In Cell 2000 platform. This analysis produced graphical profile sets that were associated with high, moderate, low or no drug-induced cellular toxicity.

High throughput electrophysiology provides a route to recording functional readouts from viable cells. The pharmaceutical industry uses PatchXpress, IonWorks and QT-screen to assess the effect of channel modulators on transgenic CHO cells overexpressing *I*_Kr_ potassium channel. Recently, it was demonstrated that high purity hPSC-cardiomyocytes could be adapted to the PatchXpress platform [79]. This allowed simultaneous recording from 16 channels and the authors quantified the effect of tetrodotoxin, nifedipine and E4031 on *I*_Na_, *I*_Ca-L_ and *I*_Kr_, respectively [79]. Further integration of hPSC-derivatives into high throughput platforms will help accelerate the use of these cells by the pharmaceutical industry.

## Conclusions and future perspectives

Recent developments have boosted the likelihood of widespread use of hPSC-derivatives in disease modelling and drug development. Reprogramming somatic cells with four genetic factors has allowed rapid derivation of many hiPSC disease models. Differentiation efficiencies have radically improved, while clinical pathologies have been demonstrably replicated in cardiac and neural hiPSC-based models. Such models respond appropriately to pharmacological challenge, particularly for LQTS and potassium or calcium channel blockers. Nevertheless, hPSC technology requires improvements. Standardised methods that stabilise the genotype, epigenome and phenotype of hPSCs and their derivatives are paramount, as are methods to quantify phenotypic responses in lineages other than hPSC-cardiomyocytes and neurons. Current differentiation methods yield heterogeneous populations of immature cells; for cardiomyocytes, this includes ventricular, atrial and pacemaker subtypes [34], but mature ventricular cells are most relevant to drug safety assessment. Although hPSCs and their derivatives are adaptable to high throughput screening, current methods are not cost effective. These are surmountable issues, especially when driven by the needs of the pharmaceutical industry, where industry figures show that 98% of sales are based on products of >5 years old. 110,000 jobs have recently been lost in the US, and patent expiry will cost the industry USD$130 during 2011–2014. Not surprisingly, most major pharmaceutical companies now have in-house stem cell programmes, and collaborate with academic groups or purchase hPSC products from commercial suppliers [39]. Just as new bioinformatics approaches are being applied to predict adverse drug interactions [80], so too will hPSC technologies in order to further understand disease and develop new drugs. Estimates indicate that even if an assay improves predictability of toxicity in humans by just 1%, up to $100 million will be saved by the pharmaceutical industry. Therefore, even small, incremental, improvements can be extremely worthwhile pursuing.

## Abbreviations

CHO: Chinese hamster ovary
hESC: human embryonic stem cell
hiPSC: human induced pluripotent stem cell
hPSC: human pluripotent stem cell
LQTS: long QT syndrome

## References

[1] Thomson JA, Itskovitz-Eldor J, Shapiro SS, Waknitz MA (1998). Embryonic stem cell lines derived from human blastocysts. Science.

[2] Yu J, Vodyanik MA, Smuga-Otto K, Antosiewicz-Bourget J (2007). Induced pluripotent stem cell lines derived from human somatic cells. Science.

[3] Takahashi K, Tanabe K, Ohnuki M, Narita M (2007). Induction of pluripotent stem cells from adult human fibroblasts by defined factors. Cell.

[4] Vazin T, Freed WJ (2010). Human embryonic stem cells: derivation, culture, and differentiation: a review. Restor Neurol Neurosci.

[5] Denning C, Allegrucci C, Priddle H, Barbadillo-Munoz MD (2006). Common culture conditions for maintenance and cardiomyocyte differentiation of the human embryonic stem cell lines, BG01 and HUES-7. Int J Dev Biol.

[6] Ludwig TE, Bergendahl V, Levenstein ME, Yu J (2006). Feeder-independent culture of human embryonic stem cells. Nat Methods.

[7] Wang L, Schulz TC, Sherrer ES, Dauphin DS (2007). Self-renewal of human embryonic stem cells requires insulin-like growth factor-1 receptor and ERBB2 receptor signaling. Blood.

[8] Mahlstedt MM, Anderson D, Sharp JS, McGilvray R (2009). Maintenance of pluripotency in human embryonic stem cells cultured on a synthetic substrate in conditioned medium. Biotechnol Bioeng.

[9] Harb N, Archer TK, Sato N (2008). The Rho-Rock-Myosin signaling axis determines cell-cell integrity of self-renewing pluripotent stem cells. PLoS One.

[10] Melkoumian Z, Weber JL, Weber DM, Fadeev AG (2010). Synthetic peptide-acrylate surfaces for long-term self-renewal and cardiomyocyte differentiation of human embryonic stem cells. Nat Biotechnol.

[11] Burridge PW, Keller G, Gold JD, Wu JC (2012). Production of de novo cardiomyocytes: human pluripotent stem cell differentiation and direct reprogramming. Cell Stem Cell.

[12] Wirth E, Lebkowski JS, Lebacqz K, Response to Frederic Bretzner (2011). Target populations for first-in-human embryonic stem cell research in spinal cord injury. Cell Stem Cell.

[13] Medina RJ, Archer DB, Stitt AW (2011). Eyes open to stem cells: safety trial may pave the way for cell therapy to treat retinal disease in patients. Stem Cell Res Ther.

[14] Skarnes WC, Rosen B, West AP, Koutsourakis M (2011). A conditional knockout resource for the genome-wide study of mouse gene function. Nature.

[15] Davis RP, van den Berg CW, Casini S, Braam SR (2011). Pluripotent stem cell models of cardiac disease and their implication for drug discovery and development. Trends Mol Med.

[16] Doevendans PA, Daemen MJ, de Muinck ED, Smits JF (1998). Cardiovascular phenotyping in mice. Cardiovasc Res.

[17] Morano I (1999). Tuning the human heart molecular motors by myosin light chains. J Mol Med (Berl).

[18] Lyons GE, Schiaffino S, Sassoon D, Barton P (1990). Developmental regulation of myosin gene expression in mouse cardiac muscle. J Cell Biol.

[19] Dubois NC, Craft AM, Sharma P, Elliott DA (2011). SIRPA is a specific cell-surface marker for isolating cardiomyocytes derived from human pluripotent stem cells. Nat Biotechnol.

[20] Bokil NJ, Baisden JM, Radford DJ, Summers KM (2010). Molecular genetics of long QT syndrome. Mol Genet Metab.

[21] Ebert AD, Yu J, Rose FF, Mattis VB (2009). Induced pluripotent stem cells from a spinal muscular atrophy patient. Nature.

[22] Devine MJ, Ryten M, Vodicka P, Thomson AJ (2011). Parkinson's disease induced pluripotent stem cells with triplication of the alpha-synuclein locus. Nat Commun.

[23] Harris A (1997). Towards an ovine model of cystic fibrosis. Hum Mol Genet.

[24] Grskovic M, Javaherian A, Strulovici B, Daley GQ (2011). Induced pluripotent stem cells – opportunities for disease modelling and drug discovery. Nat Rev Drug Discov.

[25] Mateizel I, De Temmerman N, Ullmann U, Cauffman G (2006). Derivation of human embryonic stem cell lines from embryos obtained after IVF and after PGD for monogenic disorders. Hum Reprod.

[26] Urbach A, Schuldiner M, Benvenisty N (2004). Modeling for Lesch-Nyhan disease by gene targeting in human embryonic stem cells. Stem Cells.

[27] Braam SR, Denning C, Matsa E, Young LE (2008). Feeder-free culture of human embryonic stem cells in conditioned medium for efficient genetic modification. Nat Protoc.

[28] Hockemeyer D, Wang H, Kiani S, Lai CS (2011). Genetic engineering of human pluripotent cells using TALE nucleases. Nat Biotechnol.

[29] Brennand KJ, Simone A, Jou J, Gelboin-Burkhart C (2011). Modelling schizophrenia using human induced pluripotent stem cells. Nature.

[30] Yagi T, Ito D, Okada Y, Akamatsu W (2011). Modeling familial Alzheimer's disease with induced pluripotent stem cells. Hum Mol Genet.

[31] Shi Y, Kirwan P, Smith J, Maclean G (2012). A human stem cell model of early Alzheimer's disease pathology in Down syndrome. Sci Transl Med.

[32] Marchetto MC, Carromeu C, Acab A, Yu D (2010). A model for neural development and treatment of Rett syndrome using human induced pluripotent stem cells. Cell.

[33] Carvajal-Vergara X, Sevilla A, D'Souza SL, Ang YS (2010). Patient-specific induced pluripotent stem-cell-derived models of LEOPARD syndrome. Nature.

[34] Matsa E, Rajamohan D, Dick E, Young L (2011). Drug evaluation in cardiomyocytes derived from human induced pluripotent stem cells carrying a long QT syndrome type 2 mutation. Eur Heart J.

[35] Jung CB, Moretti A, Schnitzler MM, Iop L (2011). Dantrolene rescues arrhythmogenic RYR2 defect in a patient-specific stem cell model of catecholaminergic polymorphic ventricular tachycardia. EMBO Mol Med.

[36] Yazawa M, Hsueh B, Jia X, Pasca AM (2011). Using induced pluripotent stem cells to investigate cardiac phenotypes in Timothy syndrome. Nature.

[37] Pasca SP, Portmann T, Voineagu I, Yazawa M (2011). Using iPSC-derived neurons to uncover cellular phenotypes associated with Timothy syndrome. Nat Med.

[38] Itzhaki I, Maizels L, Huber I, Zwi-Dantsis L (2011). Modelling the long QT syndrome with induced pluripotent stem cells. Nature.

[39] Braam SR, Passier R, Mummery CL (2009). Cardiomyocytes from human pluripotent stem cells in regenerative medicine and drug discovery. Trends Pharmacol Sci.

[40] Pereira GC, Silva AM, Diogo CV, Carvalho FS (2011). Drug-induced cardiac mitochondrial toxicity and protection: from doxorubicin to carvedilol. Curr Pharm Des.

[41] Hawton K, Bergen H, Waters K, Murphy E (2011). Impact of withdrawal of the analgesic Co-proxamol on nonfatal self-poisoning in the UK. Crisis.

[42] James WP, Caterson ID, Coutinho W, Finer N (2010). Effect of sibutramine on cardiovascular outcomes in overweight and obese subjects. N Engl J Med.

[43] Quigley EM (2011). Cisapride: what can we learn from the rise and fall of a prokinetic. J Dig Dis.

[44] Pouton CW, Haynes JM (2007). Embryonic stem cells as a source of models for drug discovery. Nat Rev Drug Discov.

[45] Meyer T, Leisgen C, Gonser B, Gunther E (2004). QT-screen: high-throughput cardiac safety pharmacology by extracellular electrophysiology on primary cardiac myocytes. Assay Drug Dev Technol.

[46] Dick E, Rajamohan D, Ronksley J, Denning C (2010). Evaluating the utility of cardiomyocytes from human pluripotent stem cells for drug screening. Biochem Soc Trans.

[47] May JE, Xu J, Morse HR, Avent ND (2009). Toxicity testing: the search for an in vitro alternative to animal testing. Br J Biomed Sci.

[48] Price PS, Keenan RE, Swartout JC (2008). Characterizing interspecies uncertainty using data from studies of anti-neoplastic agents in animals and humans. Toxicol Appl Pharmacol.

[49] Walton K, Dorne JL, Renwick AG (2001). Uncertainty factors for chemical risk assessment: interspecies differences in the in vivo pharmacokinetics and metabolism of human CYP1A2 substrates. Food Chem Toxicol.

[50] Holmes AM, Creton S, Chapman K (2010). Working in partnership to advance the 3Rs in toxicity testing. Toxicology.

[51] Ukelis U, Kramer PJ, Olejniczak K, Mueller SO (2008). Replacement of in vivo acute oral toxicity studies by in vitro cytotoxicity methods: opportunities, limits and regulatory status. Regul Toxicol Pharmacol.

[52] Zeevi-Levin N, Itskovitz-Eldor J, Binah O (2012). Cardiomyocytes derived from human pluripotent stem cells for drug screening. Pharmacol Ther.

[53] Nalos L, Varkevisser R, Jonsson MK, Houtman MJ (2012). Comparison of the IKr blockers moxifloxacin, dofetilide and E-4031 in five screening models of pro-arrhythmia reveals lack of specificity of isolated cardiomyocytes. Br J Pharmacol.

[54] Braam SR, Tertoolen L, van de Stolpe A, Meyer T (2010). Prediction of drug-induced cardiotoxicity using human embryonic stem cell-derived cardiomyocytes. Stem Cell Res.

[55] Young LE, Fernandes K, McEvoy TG, Butterwith SC (2001). Epigenetic change in IGF2R is associated with fetal overgrowth after sheep embryo culture. Nat Genet.

[56] Stadtfeld M, Hochedlinger K (2010). Induced pluripotency: history, mechanisms, and applications. Genes Dev.

[57] Howe SJ, Mansour MR, Schwarzwaelder K, Bartholomae C (2008). Insertional mutagenesis combined with acquired somatic mutations causes leukemogenesis following gene therapy of SCID-X1 patients. J Clin Invest.

[58] Li Y, Zhang Q, Yin X, Yang W (2011). Generation of iPSCs from mouse fibroblasts with a single gene, Oct4, and small molecules. Cell Res.

[59] Maury Y, Gauthier M, Peschanski M, Martinat C (2011). Human pluripotent stem cells for disease modelling and drug screening. BioEssays.

[60] Juopperi TA, Song H, Ming GL (2011). Modeling neurological diseases using patient-derived induced pluripotent stem cells. Future Neurol.

[61] Jonsson MK, Vos MA, Mirams GR, Duker G (2012). Application of human stem cell-derived cardiomyocytes in safety pharmacology requires caution beyond hERG. J Mol Cell Cardiol.

[62] Otsuji TG, Minami I, Kurose Y, Yamauchi K (2010). Progressive maturation in contracting cardiomyocytes derived from human embryonic stem cells: qualitative effects on electrophysiological responses to drugs. Stem Cell Res.

[63] Sartiani L, Bettiol E, Stillitano F, Mugelli A (2007). Developmental changes in cardiomyocytes differentiated from human embryonic stem cells: a molecular and electrophysiological approach. Stem Cells.

[64] Liu J, Fu JD, Siu CW, Li RA (2007). Functional sarcoplasmic reticulum for calcium handling of human embryonic stem cell-derived cardiomyocytes: insights for driven maturation. Stem Cells.

[65] Schaaf S, Shibamiya A, Mewe M, Eder A (2011). Human engineered heart tissue as a versatile tool in basic research and preclinical toxicology. PLoS One.

[66] Dengler J, Song H, Thavandiran N, Masse S (2011). Engineered heart tissue enables study of residual undifferentiated embryonic stem cell activity in a cardiac environment. Biotechnol Bioeng.

[67] Wood-Kaczmar A, Gandhi S, Yao Z, Abramov AY (2008). PINK1 is necessary for long term survival and mitochondrial function in human dopaminergic neurons. PLoS One.

[68] Steiner D, Khaner H, Cohen M, Even-Ram S (2010). Derivation, propagation and controlled differentiation of human embryonic stem cells in suspension. Nat Biotechnol.

[69] Thomas RJ, Anderson D, Chandra A, Smith NM (2009). Automated, scalable culture of human embryonic stem cells in feeder-free conditions. Biotechnol Bioeng.

[70] Desbordes SC, Placantonakis DG, Ciro A, Socci ND (2008). High-throughput screening assay for the identification of compounds regulating self-renewal and differentiation in human embryonic stem cells. Cell Stem Cell.

[71] Barbaric I, Gokhale PJ, Andrews PW (2010). High-content screening of small compounds on human embryonic stem cells. Biochem Soc Trans.

[72] Andrews PD (2011). Discovering small molecules to control stem cell fate. Future Med Chem.

[73] Kattman SJ, Witty AD, Gagliardi M, Dubois NC (2011). Stage-specific optimization of activin/nodal and BMP signaling promotes cardiac differentiation of mouse and human pluripotent stem cell lines. Cell Stem Cell.

[74] Schroeder M, Niebruegge S, Werner A, Willbold E (2005). Differentiation and lineage selection of mouse embryonic stem cells in a stirred bench scale bioreactor with automated process control. Biotechnol Bioeng.

[75] Fluri DA, Tonge PD, Song H, Baptista RP (2012). Derivation, expansion and differentiation of induced pluripotent stem cells in continuous suspension cultures. Nat Meth.

[76] Zanella F, Lorens JB, Link W (2010). High content screening: seeing is believing. Trends Biotechnol.

[77] Zock JM (2009). Applications of high content screening in life science research. Comb Chem High Throughput Screen.

[78] Foldes G, Mioulane M, Wright JS, Liu AQ (2010). Modulation of human embryonic stem cell-derived cardiomyocyte growth: a testbed for studying human cardiac hypertrophy. J Mol Cell Cardiol.

[79] Ma J, Guo L, Fiene SJ, Anson BD (2011). High purity human-induced pluripotent stem cell-derived cardiomyocytes: electrophysiological properties of action potentials and ionic currents. Am J Physiol Heart Circ Physiol.

[80] Tatonetti NP, Ye PP, Daneshjou R, Altman RB (2012). Data-driven prediction of drug effects and interactions. Sci Transl Med.

[81] Moretti A, Bellin M, Welling A, Jung CB (2010). Patient-specific induced pluripotent stem-cell models for long-QT syndrome. N Engl J Med.

[82] Lahti AL, Kujala VJ, Chapman H, Koivisto AP (2011). Human disease model for long QT syndrome type 2 using iPS cells demonstrates arrhythmogenic characteristics in cell culture. Dis Model Mech.

[83] Fatima A, Xu G, Shao K, Papadopoulos S (2011). In vitro modeling of ryanodine receptor 2 dysfunction using human induced pluripotent stem cells. Cell Physiol Biochem.

[84] Liu GH, Barkho BZ, Ruiz S, Diep D (2011). Recapitulation of premature ageing with iPSCs from Hutchinson-Gilford progeria syndrome. Nature.

[85] Zhang J, Lian Q, Zhu G, Zhou F (2010). A human iPSC model of Hutchinson Gilford Progeria reveals vascular smooth muscle and mesenchymal stem cell defects. Cell Stem Cell.

[86] Park IH, Arora N, Huo H, Maherali N (2008). Disease-specific induced pluripotent stem cells. Cell.

[87] Dick E, Matsa E, Bispham J, Reza M (2011). Two new protocols to enhance the production and isolation of human induced pluripotent stem cell lines. Stem Cell Res.

[88] Kazuki Y, Hiratsuka M, Takiguchi M, Osaki M (2010). Complete genetic correction of ips cells from Duchenne muscular dystrophy. Mol Ther.

[89] Song B, Sun G, Herszfeld D, Sylvain A (2012). Neural differentiation of patient specific iPS cells as a novel approach to study the pathophysiology of multiple sclerosis. Stem Cell Res.

[90] Chamberlain SJ, Chen P-F, Ng KY, Bourgois-Rocha F (2010). Induced pluripotent stem cell models of the genomic imprinting disorders Angelman and Prader-Willi syndromes. Proc Natl Acad Sci USA.

[91] Yang J, Cai J, Zhang Y, Wang X (2010). Induced pluripotent stem cells can be used to model the genomic imprinting disorder Prader-Willi syndrome. J Biol Chem.

[92] Tolar J, Xia L, Riddle MJ, Lees CJ (2010). Induced pluripotent stem cells from individuals with recessive dystrophic epidermolysis bullosa. J Invest Dermatol.

[93] Chang T, Zheng W, Tsark W, Bates SE (2011). Phenotypic rescue of induced pluripotent stem cell-derived motoneurons of a spinal muscular atrophy patient. Stem Cells.

[94] Lee G, Papapetrou EP, Kim H, Chambers SM (2009). Modelling pathogenesis and treatment of familial dysautonomia using patient-specific iPSCs. Nature.

[95] Cheung AY, Horvath LM, Grafodatskaya D, Pasceri P (2010). Isolation of MECP2-null Rett Syndrome patient hiPS cells and isogenic controls through X-chromosome inactivation. Hum Mol Genet.

[96] Amenduni M, De Filippis R, Cheung AY, Disciglio V (2011). iPS cells to model CDKL5-related disorders. Eur J Hum Genet.

[97] Chiang CH, Su Y, Wen Z, Yoritomo N (2011). Integration-free induced pluripotent stem cells derived from schizophrenia patients with a DISC1 mutation. Mol Psychiatry.

[98] Yahata N, Asai M, Kitaoka S, Takahashi K (2011). Anti-abeta drug screening platform using human iPS cell-derived neurons for the treatment of Alzheimer's disease. PLoS One.

[99] Soldner F, Hockemeyer D, Beard C, Gao Q (2009). Parkinson's disease patient-derived induced pluripotent stem cells free of viral reprogramming factors. Cell.

[100] Seibler P, Graziotto J, Jeong H, Simunovic F (2011). Mitochondrial Parkin recruitment is impaired in neurons derived from mutant PINK1 induced pluripotent stem cells. J Neurosci.

[101] Sanchez-Danes A, Richaud-Patin Y, Carballo-Carbajal I, Jimenez-Delgado S (2012). Disease-specific phenotypes in dopamine neurons from human iPS-based models of genetic and sporadic Parkinson's disease. EMBO Mol Med.

[102] Sheridan SD, Theriault KM, Reis SA, Zhou F (2012). Epigenetic characterization of the FMR1 gene and aberrant neurodevelopment in human induced pluripotent stem cell models of fragile X syndrome. PLoS One.

[103] Liu J, Verma PJ, Evans-Galea MV, Delatycki MB (2011). Generation of induced pluripotent stem cell lines from Friedreich ataxia patients. Stem Cell Rev.

[104] Zhang N, An MC, Montoro D, Ellerby LM (2010). Characterization of human Huntington's disease cell model from induced pluripotent stem cells. PLoS Curr.

[105] Luo Y, Fan Y, Zhou B, Xu Z (2012). Generation of induced pluripotent stem cells from skin fibroblasts of a patient with olivopontocerebellar atrophy. Tohoku J Exp Med.

[106] Derosa BA, Van Baaren JM, Dubey GK, Vance JM (2012). Derivation of autism spectrum disorder-specific induced pluripotent stem cells from peripheral blood mononuclear cells. Neurosci Lett.

[107] Dimos JT, Rodolfa KT, Niakan KK, Weisenthal LM (2008). Induced pluripotent stem cells generated from patients with ALS can be differentiated into motor neurons. Science.

[108] Maehr R, Chen S, Snitow M, Ludwig T (2009). Generation of pluripotent stem cells from patients with type 1 diabetes. Proc Natl Acad Sci USA.

[109] Ohmine S, Squillace KA, Hartjes KA, Deeds MC (2012). Reprogrammed keratinocytes from elderly type 2 diabetes patients suppress senescence genes to acquire induced pluripotency. Aging (Albany NY).

[110] Rashid ST, Corbineau S, Hannan N, Marciniak SJ (2010). Modeling inherited metabolic disorders of the liver using human induced pluripotent stem cells. J Clin Invest.

[111] Sebastiano V, Maeder ML, Angstman JF, Haddad B (2011). In situ genetic correction of the sickle cell anemia mutation in human induced pluripotent stem cells using engineered zinc finger nucleases. Stem Cells.

[112] Zou J, Mali P, Huang X, Dowey SN (2011). Site-specific gene correction of a point mutation in human iPS cells derived from an adult patient with sickle cell disease. Blood.

[113] Raya A, Rodriguez-Piza I, Guenechea G, Vassena R (2009). Disease-corrected haematopoietic progenitors from Fanconi anaemia induced pluripotent stem cells. Nature.

[114] Muller LU, Milsom MD, Harris CE, Vyas R (2012). Overcoming reprogramming resistance of Fanconi anemia cells. Blood.

[115] Ye Z, Zhan H, Mali P, Dowey S (2009). Human-induced pluripotent stem cells from blood cells of healthy donors and patients with acquired blood disorders. Blood.

[116] Wang Y, Jiang Y, Liu S, Sun X (2009). Generation of induced pluripotent stem cells from human beta-thalassemia fibroblast cells. Cell Res.

[117] Wang Y, Zheng CG, Jiang Y, Zhang J (2012). Genetic correction of beta-thalassemia patient-specific iPS cells and its use in improving hemoglobin production in irradiated SCID mice. Cell Res.

[118] Jin ZB, Okamoto S, Osakada F, Homma K (2011). Modeling retinal degeneration using patient-specific induced pluripotent stem cells. PLoS One.

[119] Howden SE, Gore A, Li Z, Fung HL (2011). Genetic correction and analysis of induced pluripotent stem cells from a patient with gyrate atrophy. Proc Natl Acad Sci USA.

[120] Qiu X, Yang J, Liu T, Jiang Y (2012). Efficient generation of lens progenitor cells from cataract patient-specific induced pluripotent stem cells. PLoS One.

[121] Agarwal S, Loh YH, McLoughlin EM, Huang J (2010). Telomere elongation in induced pluripotent stem cells from dyskeratosis congenita patients. Nature.

[122] Xue T, Cho HC, Akar FG, Tsang SY (2005). Functional integration of electrically active cardiac derivatives from genetically engineered human embryonic stem cells with quiescent recipient ventricular cardiomyocytes. Circulation.

[123] Norstrom A, Akesson K, Hardarson T, Hamberger L (2006). Molecular and pharmacological properties of human embryonic stem cell-derived cardiomyocytes. Exp Biol Med (Maywood).

[124] Yokoo N, Baba S, Kaichi S, Niwa A (2009). The effects of cardioactive drugs on cardiomyocytes derived from human induced pluripotent stem cells. Biochem Biophys Res Commun.

[125] Kang J, Chen XL, Ji J, Lei Q (2012). Ca^2+^ channel activators reveal differential L-type Ca^2+^ channel pharmacology between native and stem cell-derived cardiomyocytes. J Pharmacol Exp Ther.

[126] Liu J, Lieu DK, Siu CW, Fu JD (2009). Facilitated maturation of Ca^2+^ handling properties of human embryonic stem cell-derived cardiomyocytes by calsequestrin expression. Am J Physiol Cell Physiol.

[127] Germanguz I, Sedan O, Zeevi-Levin N, Shtreichman R (2009). Molecular characterization and functional properties of cardiomyocytes derived from human inducible pluripotent stem cells. J Cell Mol Med.

[128] Zwi L, Caspi O, Arbel G, Huber I (2009). Cardiomyocyte differentiation of human induced pluripotent stem cells. Circulation.

[129] Mummery C, Ward-van Oostwaard D, Doevendans P, Spijker R (2003). Differentiation of human embryonic stem cells to cardiomyocytes: role of coculture with visceral endoderm-like cells. Circulation.

[130] Brito Martins M, Harding S, Ali N (2008). β1- and β2-Adrenoceptor responses in cardiomyocytes derived from human embryonic stem cells: comparison with failing and non-failing adult human heart. Br J Pharmacol.

[131] Kehat I, Kenyagin-Karsenti D, Snir M, Segev H (2001). Human embryonic stem cells can differentiate into myocytes with structural and functional properties of cardiomyocytes. J Clin Invest.

[132] Honda M, Kiyokawa J, Tabo M, Inoue T (2011). Electrophysiological characterization of cardiomyocytes derived from human induced pluripotent stem cells. J Pharmacol Sci.

[133] Xu C, Police S, Rao N, Carpenter MK (2002). Characterization and enrichment of cardiomyocytes derived from human embryonic stem cells. Circ Res.

[134] Satin J, Kehat I, Caspi O, Huber I (2004). Mechanism of spontaneous excitability in human embryonic stem cell derived cardiomyocytes. J Physiol.

[135] Guo L, Qian JY, Abrams R, Tang HM (2011). The electrophysiological effects of cardiac glycosides in human iPSC-derived cardiomyocytes and in guinea pig isolated hearts. Cell Physiol Biochem.

[136] Jonsson MK, Duker G, Tropp C, Andersson B (2010). Quantified proarrhythmic potential of selected human embryonic stem cell-derived cardiomyocytes. Stem Cell Res.

[137] Pekkanen-Mattila M, Chapman H, Kerkela E, Suuronen R (2010). Human embryonic stem cell-derived cardiomyocytes: demonstration of a portion of cardiac cells with fairly mature electrical phenotype. Exp Biol Med (Maywood).

[138] Tanaka T, Tohyama S, Murata M, Nomura F (2009). In vitro pharmacologic testing using human induced pluripotent stem cell-derived cardiomyocytes. Biochem Biophys Res Commun.

[139] He JQ, Ma Y, Lee Y, Thomson JA (2003). Human embryonic stem cells develop into multiple types of cardiac myocytes. Circ Res.

[140] Zhang J, Wilson GF, Soerens AG, Koonce CH (2009). Functional cardiomyocytes derived from human induced pluripotent stem cells. Circ Res.

[141] Burridge PW, Thompson S, Millrod MA, Weinberg S (2011). A universal system for highly efficient cardiac differentiation of human induced pluripotent stem cells that eliminates interline variability. PLoS One.

[142] Itzhaki I, Rapoport S, Huber I, Mizrahi I (2011). Calcium handling in human induced pluripotent stem cell derived cardiomyocytes. PLoS One.

[143] Yang L, Soonpaa MH, Adler ED, Roepke TK (2008). Human cardiovascular progenitor cells develop from a KDR+ embryonic-stem-cell-derived population. Nature.

[144] Dolnikov K, Shilkrut M, Zeevi-Levin N, Danon A (2005). Functional properties of human embryonic stem cell-derived cardiomyocytes. Ann N Y Acad Sci.

[145] Reppel M, Pillekamp F, Brockmeier K, Matzkies M (2005). The electrocardiogram of human embryonic stem cell-derived cardiomyocytes. J Electrocardiol.

[146] Cohen J, Babiarz J, Abrams R, Guo L (2011). Use of human stem cell derived cardiomyocytes to examine sunitinib mediated cardiotoxicity and electrophysiological alterations. Toxicol Appl Pharmacol.

[147] Yoon BS, Yoo SJ, Lee JE, You S (2006). Enhanced differentiation of human embryonic stem cells into cardiomyocytes by combining hanging drop culture and 5-azacytidine treatment. Differentiation.

[148] Cao N, Liu Z, Chen Z, Wang J (2012). Ascorbic acid enhances the cardiac differentiation of induced pluripotent stem cells through promoting the proliferation of cardiac progenitor cells. Cell Res.

[149] Pal R, Mamidi MK, Das AK, Bhonde R (2012). Diverse effects of dimethyl sulfoxide (DMSO) on the differentiation potential of human embryonic stem cells. Arch Toxicol.

[150] Schuldiner M, Yanuka O, Itskovitz-Eldor J, Melton DA (2000). Effects of eight growth factors on the differentiation of cells derived from human embryonic stem cells. Proc Natl Acad Sci USA.

[151] Fujiwara M, Yan P, Otsuji TG, Narazaki G (2011). Induction and enhancement of cardiac cell differentiation from mouse and human induced pluripotent stem cells with cyclosporin-A. PLoS One.

[152] Gaur M, Ritner C, Sievers R, Pedersen A (2010). Timed inhibition of p38MAPK directs accelerated differentiation of human embryonic stem cells into cardiomyocytes. Cytotherapy.

[153] Graichen R, Xu X, Braam SR, Balakrishnan T (2008). Enhanced cardiomyogenesis of human embryonic stem cells by a small molecular inhibitor of p38 MAPK. Differentiation.

[154] Hudson JE, Zimmermann WH (2011). Tuning Wnt-signaling to enhance cardiomyogenesis in human embryonic and induced pluripotent stem cells. J Mol Cell Cardiol.

[155] Willems E, Spiering S, Davidovics H, Lanier M (2011). Small-molecule inhibitors of the Wnt pathway potently promote cardiomyocytes from human embryonic stem cell-derived mesoderm. Circ Res.

[156] Paige SL, Osugi T, Afanasiev OK, Pabon L (2010). Endogenous Wnt/beta-catenin signaling is required for cardiac differentiation in human embryonic stem cells. PLoS One.

[157] Blin G, Nury D, Stefanovic S, Neri T (2010). A purified population of multipotent cardiovascular progenitors derived from primate pluripotent stem cells engrafts in postmyocardial infarcted nonhuman primates. J Clin Invest.

[158] Zhang Q, Jiang J, Han P, Yuan Q (2011). Direct differentiation of atrial and ventricular myocytes from human embryonic stem cells by alternating retinoid signals. Cell Res.

[159] Oldershaw RA, Baxter MA, Lowe ET, Bates N (2010). Directed differentiation of human embryonic stem cells toward chondrocytes. Nat Biotechnol.

[160] Xu C, Police S, Hassanipour M, Li Y (2011). Efficient generation and cryopreservation of cardiomyocytes derived from human embryonic stem cells. Regen Med.

[161] Takei S, Ichikawa H, Johkura K, Mogi A (2009). Bone morphogenetic protein-4 promotes induction of cardiomyocytes from human embryonic stem cells in serum-based embryoid body development. Am J Physiol Heart Circ Physiol.

